# A cyst-forming coccidian with large geographical range infecting forest and commensal rodents: *Sarcocystis muricoelognathis* sp. nov.

**DOI:** 10.1186/s13071-024-06230-8

**Published:** 2024-03-15

**Authors:** Tao Qin, Paula Ortega-Perez, Gudrun Wibbelt, Maklarin B. Lakim, Sulaiman Ginting, Yuvaluk Khoprasert, Konstans Wells, Junjie Hu, Thomas Jäkel

**Affiliations:** 1 School of Ecology and Environmental Sciences and Yunnan International Joint Laboratory of Virology & Immunity, Yunnan University, Kunming, China; 2 Department of Pathology, AnaPath Services GmbH, Liestal, Switzerland; 3 Department Wildlife Diseases, Leibniz Institute for Zoo and Wildlife Research, Berlin, Germany; 4 Sabah Parks, Kota Kinabalu, Sabah Malaysia; 5 Islamic University of North Sumatra, Medan, Indonesia; 6 Department of Agriculture, Plant Protection Research and Development Office, Bangkok, Thailand; 7 Department of Biosciences, Swansea University, Swansea, UK; 8 Institute of Biology, Department of Parasitology, University of Hohenheim, Stuttgart, Germany

**Keywords:** *Rattus norvegicus*, *Maxomys whiteheadi*, *Coelognathus radiatus*, *Coelognathus flavolineatus*, *Sarcocystis muricoelognathis*, Life cycle, Morphological and molecular characterization, *Sarcocystis zuoi*-complex

## Abstract

**Background:**

The geographic distribution and host-parasite interaction networks of *Sarcocystis* spp. in small mammals in eastern Asia remain incompletely known.

**Methods:**

Experimental infections, morphological and molecular characterizations were used for discrimination of a new *Sarcocystis* species isolated from colubrid snakes and small mammals collected in Thailand, Borneo and China.

**Results:**

We identified a new species, *Sarcocystis muricoelognathis* sp. nov., that features a relatively wide geographic distribution and infects both commensal and forest-inhabiting intermediate hosts. *Sarcocystis* sporocysts collected from rat snakes (*Coelognathus radiatus*, *C. flavolineatus*) in Thailand induced development of sarcocysts in experimental SD rats showing a type 10a cyst wall ultrastructure that was identical with those found in *Rattus norvegicus* from China and the forest rat *Maxomys whiteheadi* in Borneo. Its cystozoites had equal sizes in all intermediate hosts and locations, while sporocysts and cystozoites were distinct from other *Sarcocystis* species. Partial *28S rRNA* sequences of *S*. *muricoelognathis* from *M. whiteheadi* were largely identical to those from *R. norvegicus* in China but distinct from newly sequenced *Sarcocystis* *zuoi*. The phylogeny of the nuclear *18S rRNA* gene placed *S. muricoelognathis* within the so-called *S. zuoi* complex, including *Sarcocystis* *attenuati*, *S*. *kani*, *S*. *scandentiborneensis* and *S*. *zuoi*, while the latter clustered with the new species. However, the phylogeny of the ITS1-region confirmed the distinction between *S*. *muricoelognathis* and *S*. *zuoi*. Moreover, all three gene trees suggested that an isolate previously addressed as *S*. *zuoi* from Thailand (KU341120) is conspecific with *S*. *muricoelognathis*. Partial mitochondrial *cox1* sequences of *S. muricoelognathis* were almost identical with those from other members of the group suggesting a shared, recent ancestry. Additionally, we isolated two partial *28S rRNA Sarcocystis* sequences from Low’s squirrel *Sundasciurus lowii* that clustered with those of *S*. *scandentiborneensis* from treeshews.

**Conclusions:**

Our results provide strong evidence of broad geographic distributions of rodent-associated *Sarcocystis* and host shifts between commensal and forest small mammal species, even if the known host associations remain likely only snapshots of the true associations.

**Graphical Abstract:**

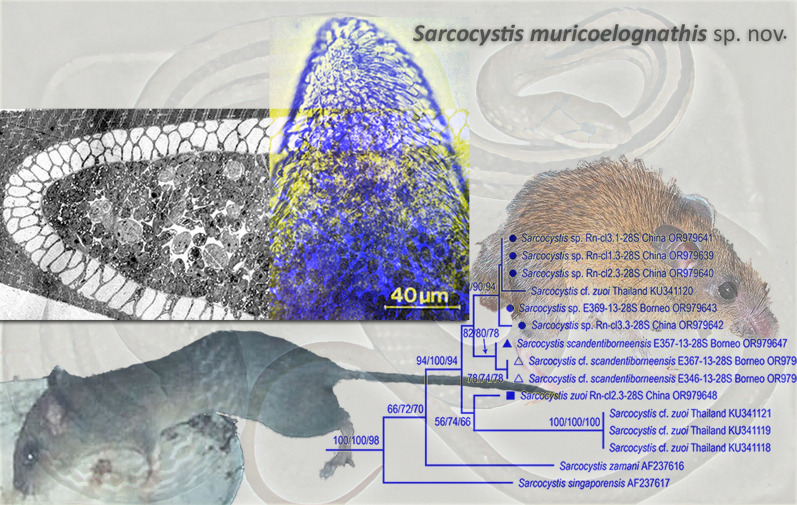

**Supplementary Information:**

The online version contains supplementary material available at 10.1186/s13071-024-06230-8.

## Background

*Sarcocystis* spp. of rodents represent a highly diversified group of tissue cyst-forming coccidia, for which the patterns of species diversification, geographical distribution and host associations remain poorly known. Over the last 5 decades, various species of *Sarcocystis* have been observed in the striated musculature of commensal *Rattus* spp. and related rodent species, including the widespread *Sarcocystis cymruensis* [1–5], *S. kani* [6], *S. murinotechis* [7], *S. ratti* [5, 8], *S. singaporensis*, *S. villivillosi*, *S. zamani* [2, 9], *S. sulawesiensis* [2, 10], *S. zuoi* [11] and a *Sarcocystis* sp. from masked owls [12]. *Sarcocystis rodentifelis* [13, 14] is considered synonymous with *S*. *cymruensis* [3]. In this group, *Sarcocystis* spp. with snake-rodent life cycle are among the most prevalent Apicomplexan parasites found in rodents in the Indo-Australian Archipelago [2, 10, 15], also showing prominent presence in environmental samples [16].

*Sarcocystis murinotechis* has been a somewhat enigmatic parasite, because since its early description from infected elapid snakes (*Notechis ater*; now, *Notechis scutatus* [Peters, 1861], see [17]) and rodents in Australia, i.e. *Rattus rattus*, *R. norvegicus*, *R. lutreolus*, *Pseudomys higginsi* and *Mastacomys fuscus* [7, 18], no molecular information has become available. Furthermore, the ultrastructure of its sarcocyst shares some similarities with *Sarcocystis* spp. from Eastern and Southeast Asia, such as *S. zuoi* from China, which develops in colubrid snakes as definitive host [11], and a species described as *S*. *murinotechis*-like that was observed in rats in Thailand and neighboring countries [2, 15]. Thus, more morphological as well as molecular data were needed to discriminate between these species. Watthanakaiwan et al. [19] amplified nuclear *rRNA* gene sequences from a *Sarcocystis* species observed in *Rattus tiomanicus* in Thailand, which appeared to be molecularly close to *S. zuoi* and was subsequently cited as this species although the authors did not provide morphological evidence for its cyst wall structure. In a recent study, we found that one of the *18S rRNA* gene sequences (KU341120) published by Watthanakaiwan et al. [19] was genetically distinct from *S. zuoi* and hypothesized that it could belong to another species [20].

Here, we synthesized comparative morphological and molecular evidence from new and previously collected specimens from Thailand, Borneo and China and insights from transmission experiments using sporocysts from naturally infected colubrid (rat) snakes in Thailand to clarify the status of the *S. murinotechis*-like species in Thailand and its potential distribution in other countries of the region. We describe a new *Sarcocystis* species and discuss its phylogenetic position and host relationships within the so-called *S. zuoi* complex, which is an expanding group of molecularly cryptic *Sarcocystis* spp. that infects colubrid snakes and small mammals in Asia [6].

## Methods

### Sample collection and examination

*Sarcocystis* oocysts/sporocysts were recovered from the intestines of two road-killed, adult colubrid snakes, one copperhead rat snake, *Coelognathus radiatus*, collected in the Bangkok metropolitan area, and one yellow striped snake, *Coelognathus flavolineatus*, which had been collected in Chumphon Province in southern Thailand. Intestinal scrapings containing sporocysts were further purified on a discontinuous sucrose density gradient as described previously [6].

The rodents from Borneo examined for this study included two *Maxomys whiteheadi*, three *Niviventer cremoriventer* and four *Sundasciurus lowii* that were live-trapped between 2012 and 2013 using locally made drop-door, wire-mesh traps in different habitat types from mature forest to urban habitats in the Kota Kinabalu and Ranau Districts in Sabah, Malaysia. Species identification was based on phenotypic characteristics according to Aplin et al. [21] and Musser and Carleton [22]. Captured animals were transferred into small cotton bags and killed by cervical dislocation. Samples of striated musculature from diaphragm and latero-ventral abdominal wall (Musculus obliquus externus) or M. quadriceps femoris were fixed and stored in 70% ethanol. Histological preparation of sarcocysts in muscle samples and subsequent processing for ultrastructural examination have been described [20].

In China, a total of 189 Norway rats were captured between 2017 and 2021 by live trapping on farmland in Anning and Nanjian Prefectures, both located in southwestern China. After the animals had been killed with CO_2_, their corpses were examined in a laboratory for presence of sarcocysts. This included light microscopical inspection of fresh preparations of musculature from tongue, esophagus, diaphragm, various skeletal muscles and the heart. Single sarcocysts were separated from surrounding tissue using dissection needles and subsequently processed for transmission electron microscopy (TEM) as described [23].

### Light microscopic size measurements of parasite stages and statistical analysis

The sizes of sporocysts and cystozoites of the new *Sarcocystis* isolates under study were measured and compared with other species by using one-way ANOVA as previously described [6].

### Husbandry and experimental infection of laboratory rats

All experiments involving the transmission of *Sarcocystis* sporocysts to a rodent host were performed using outbred Sprague-Dawley (SD) rats, which were obtained from the National Laboratory Animal Centre of Mahidol University in Thailand. SD rats were kept under coccidia-free conditions in isolated and climate-controlled rooms of the Agricultural Zoology Research Group at the Department of Agriculture in Bangkok, Thailand. Two individuals were housed together per cage (Makrolon polycarbonate: 60 × 38 × 20 cm) under controlled temperature and humidity conditions, exposed to a 12:12 h light/dark cycle and ad libitum diet and water supply.

Sporocysts isolated from one *Coelognathus radiatus* and one *C. flavolineatus* were used to inoculate three male, 3-month-old SD rats each per isolate; additionally, two rats each were inoculated with PBS and kept in the same room serving as negative controls. The animals were inoculated with about 1000 purified sporocysts each using a stomach tube. Afterwards, the animals were monitored daily for potential signs of disease. All six parasite-inoculated rats and the four negative control rats were killed after 5 months with CO_2_ and necropsied. We examined fresh muscle tissue from esophagus, tongue, diaphragm, muscles of the abdominal wall, femoral muscles and masseter for presence of sarcocysts under a light microscope. The size of freshly released cystozoites was determined after rupturing individual sarcocysts in phosphate-buffered saline at room temperature. Samples of muscle tissue were fixed and processed for electron microscopy as described previously [24].

### Gene sequencing and sequence comparisons using BLAST

Only samples from Borneo and China were available for molecular studies, as samples taken during the laboratory infection experiments in Thailand had been destroyed during storage. For samples from Borneo, DNA extraction was performed as described in detail previously [20]; 25 µg samples of striated musculature were used that had been checked for presence of sarcocysts by LM and TEM. Nuclear *28S rRNA* gene sequences were amplified following the protocol of Prakas et al. [25] using the primers KL-P1F and KL-P1R (Table 1). Raw sequences were manually checked and refined using MEGA-6 [26]. For samples from China, four sarcocysts derived from different individual Norway rats were used for molecular analysis. DNA was extracted from each sarcocyst using a commercial kit (TIanamp Genomic DNA, Tiangen Biotech^®^, Beijing, China) according to the manufacturer’s instructions. Four genes, nuclear *18S rRNA*, *28S rRNA*, ITS1*-5.8S-*ITS-2 and mitochondrial *cox1*, were selected for the characterization of this species. The primers used are listed in Table 1. PCR amplifications were performed as previously described [23]. PCR products were gel purified using an E.Z.N.A.^®^ Gel Extraction Kit (Omega Bio-Tek, Inc., Georgia, USA) and ligated to pCE2 TA/Blunt-Zero vector using a 5-min TA/Blunt-Zero Cloning Kit (Vazyme Biotech Co., Ltd., Nanjing, China) according to the product manuals. The ligated vectors were transformed to Trelief^®^ 5α Chemically Competent Cell (Tsingke Biotechnology Co., Ltd., Beijing, China). The positive bacterial colonies were selected for bi-directional sequencing on an ABI PRISM TM 3730 XL DNA Analyzer.

**Table 1:** Primers used for PCR amplification of nuclear and mitochondrial genes of the novel *Sarcocystis* species

DNA region	Primer name	Primer sequence (5′–3′)	References
Samples from China			
*18S rRNA*	ERIB1^a^	ACCTGGTTGATCCTGCCAG	[66]
	S2^b^	CTGATCGTCTTCGAGCCCCTA	[67]
	S3^a^	TTGTTAAAGACGAACTACTGCG	[67]
	B^b^	GATCCTTCTGCAGGTTCACCTAC	[68]
*28S rRNA*	KL1^a^	TACCCGCTGAACTTAAGC	[69]
	KL3^b^	CCACCAAGATCTGCACTAG	[69]
	Sarm28S^a^	TCGCTCATCAGATACCA	This study
	Sarm28S^b^	ATTGCGTCAACACCAG	This study
	KL6a^a^	GGATTGGCTCTGAGGG	[69]
	KL2^b^	ACTTAGAGGCGTTCAGTC	[69]
ITS1-*5.8S*-ITS2	SarmITSF^a^	ACGTCCCTGCCCTTTGTA	This study
	SarmITSR^b^	ACTTCTCCCTRTGCCTCC	This study
*cox1*	526F1^a^	TCCTTCCTGGCGTACAACAAT CAT	[23]
	1209R1^b^	GGGGCATGACATTGAAAGCAAGTA	[23]
Samples from Borneo			
*28S rRNA*	KL-P1F^a^	TACCCGCTGAACTTAAGCAT	[25]
	KL-P1R^b^	CCCAAGTTTGACGAACGATT	[25]

^a^Forward primer

^b^Reverse primer

Initial GenBank screening and pairwise similarity comparisons of the amplified sequences were performed using the web-based Basic Local Alignment Search Tool (BLASTn) of the National Center for Biotechnology Information of the National Institutes of Health, USA.

### Phylogenetic analysis

#### Alignment of rRNA sequences and identification of gene boundaries

Before alignment of the *18S* and *28S rRNA* sequences and the Internal Transcribed Spacer 1 (ITS1), we identified their gene boundaries based on the predicted secondary structure of the rRNA of *Toxoplasma gondii* as published by Gagnon et al. [27] and truncated the ends if necessary. For the *18S rRNA* gene, we additionally employed secondary structure modeling using SSU-ALIGN software (version 0.1.1, February 2016) as described previously [6]. Full-length *28S rRNA* sequences were truncated from the 3’-end to a maximum of 2500 nucleotides to accommodate the analysis limits of the alignment software.

The new ITS1 sequences described here were carefully inspected and excised from the amplified ITS1*-5.8S-*ITS2 gene complex. For sequence comparisons, 25 Apicomplexan ITS1 sequences were selected that were labeled as being complete by GenBank. Especially, we checked whether they started after the *18 s rRNA* [28] and were consistent with the start motifs that followed the terminal motif (3ʹ-end) of the nuclear *18S rRNA* gene of *T. gondii*: 5ʹ-TCATTCA-3ʹ. After this motif, ITS1 sequences typically started with 5ʹ-CACA-3ʹ (e.g. *Sarcocystis* cf. *zuoi*, KU341120), 5ʹ-AACG-3ʹ (e.g. *S*. *neurona*, AF081944), 5ʹ-CACC-3ʹ (e.g. *S*. *hirsuta*, KT901231) and 5ʹ-CACG-3ʹ (e.g. *T. gondii*, AY259045), or similar. To identify the terminal nucleotides of an ITS1 sequence, we searched for the potential presence of the start motif of the *5.8S rRNA* of *T. gondii* (5ʹ-AAATTTT’-3ʹ) according to Gagnon et al. [27], which was slightly modified in *Sarcocystis* spp. to 5ʹ-ACAATTTT-3ʹ and included in a stretch of about 230 nucleotides (nt) of the highly conserved *5.8S rRNA* gene. Accordingly, we truncated all newly sequenced ITS1 sequences before this start motif, which resulted in ITS1 sequences that were 629–675 nt long. We did not alter the ends of the other, publicly available ITS1 sequences of tissue-cyst forming coccidia, which were analyzed as they were, except in the case of the four ITS1 sequences that had been designated as ‘*Sarcocystis zuoi*’ from Thailand (KU341118/19/20/21) and deposited as gene complex, requiring excision for further alignment.

The *18S*, *28S rRNA* and ITS1 sequences of the *Sarcocystis* isolates under investigation were aligned with 38, 25 and 25 published Apicomplexan sequences available at GenBank [29], respectively, using the multiple sequence alignment algorithm of the ‘R-Coffee’ web server, which considers the predicted secondary structure of RNA [30]. The alignment procedure was repeated several times, while we finally selected three independent alignments with the smallest proportion of ambiguous positions for each gene. Conserved areas were inspected visually for potential misalignments.

#### Phylogenetic reconstruction of gene trees

Reconstruction of phylogenetic trees in the case of the *18S* and *28S rRNA* genes was performed by the maximum likelihood (ML) and minimum evolution (ME) methods as implemented in Mega-X [31]. The ME criterion was used because it provided a distance-based approach to tree-building in contrast to the character-based ML [32]. As implemented in Mega-X, ME also allowed for distance estimation under heterogeneous substitution patterns among lineages [33], a condition that applies to coccidian parasites [34]. Our initial analyses of the *18S* had shown that ML could not resolve (into dichotomies) the clade containing the new *Sarcocystis* species and *S*. *zuoi*. Initial tree(s) for the heuristic search under ML were obtained automatically by applying neighbor-joining and BioNJ algorithms. The ME tree was searched using the close-neighbor-interchange (CNI) algorithm at a search level of 2; the neighbor-joining algorithm was used to generate the initial tree. For analysis of the ITS1 locus, initial tree(s) were obtained by applying neighbor-joining and BioNJ algorithms to a matrix of pairwise distances estimated using the maximum composite likelihood (MCL) approach. For all analyses, 1000 bootstrap replicates were performed.

The appropriate models for nucleotide substitution rates were determined by ML analysis using MEGA-X, selecting for each gene the model with the lowest BIC (Bayesian information criterion) and AICc (Akaike information criterion, corrected) values. For analysis of a longer stretch of the nuclear *28S rRNA* gene (comparison of 1300–1400 homologous characters), the Tamura-Nei substitution model was used with a discrete gamma distribution rate variation (four categories; + G, parameter = 0.6874) while the rate variation model allowed for some sites to be invariable (+ I; 32.39% of sites). For shorter *28S rRNA* sequences (360–400 characters), the Kimura two-parameter model was employed with a discrete gamma distribution (four categories; + G, parameter = 0.2299). For the nuclear *18S rRNA* gene under ML analysis, we selected the Tamura three-parameter substitution model with a discrete gamma distribution (four categories; + G, parameter = 0.1537) and some sites being evolutionarily invariable ([+ I], 38.77% sites). For ME phylogenetic analysis of the *18S rRNA*, the rate variation among sites was modeled with a gamma distribution (shape parameter = 0.16); the differences in the composition bias among sequences were considered in evolutionary comparisons. The ITS1 sequences were analyzed using the Hasegawa-Kishino-Yano substitution model under a discrete gamma distribution to model evolutionary rate differences among sites (four categories; + G, parameter = 4.2949).

For comparison of other *Sarcocystis* spp. with the novel *Sarcocystis* isolates, we included all sequences in tree building that showed a high degree of sequence similarity during initial GenBank screens with BLASTn. Second, we selected sequences of known *Sarcocystis* spp. parasitizing snakes as definitive hosts and representative taxa of major lineages of the Sarcocystinae and Toxoplasmatinae as described [6, 35]. Eimeriid coccidia served as outgroup. Accession numbers of all sequences are given in the phylogenetic trees of the respective genes.

## Results

### Sarcocyst development in experimentally infected rats

**Fig. 1:**
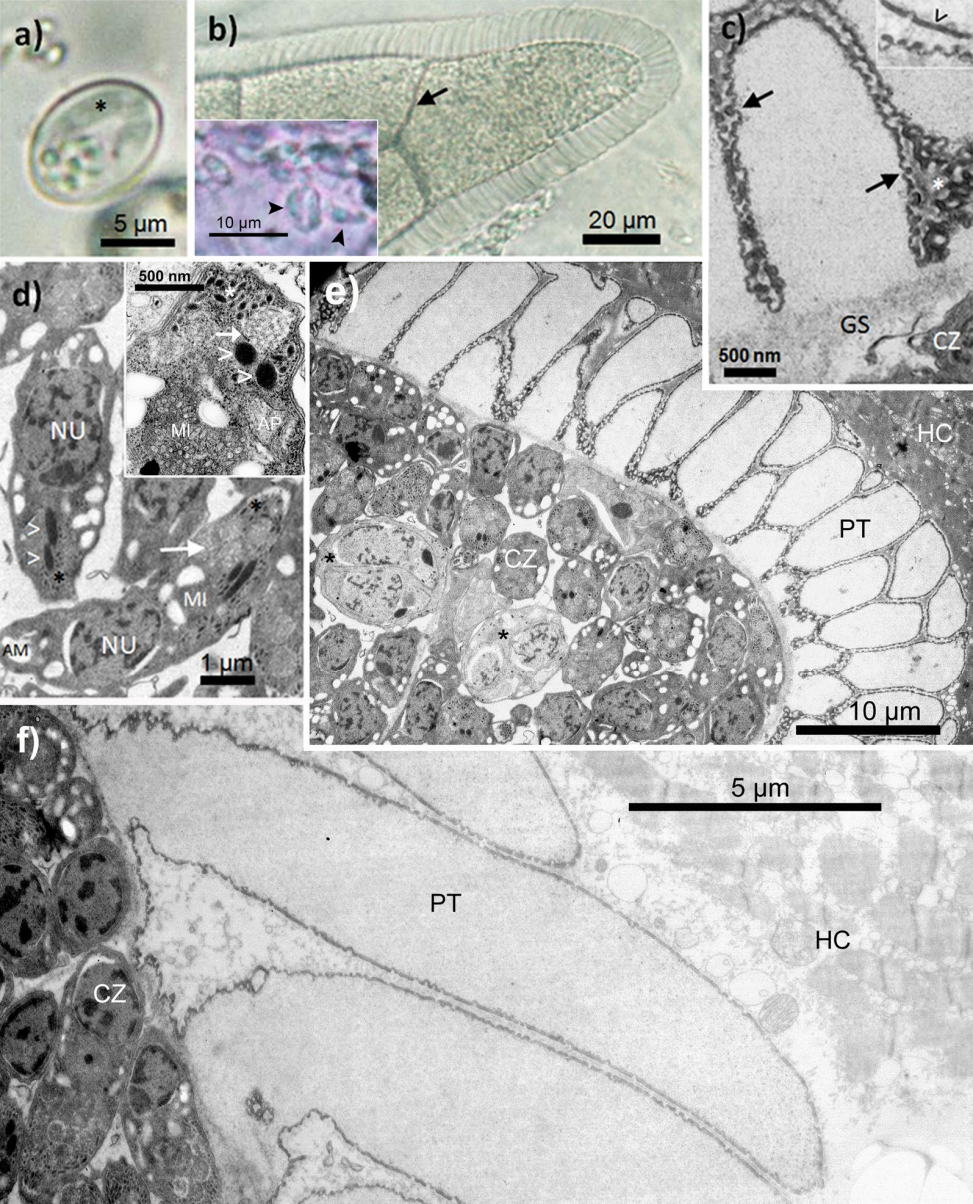
**a**–**f** Light microscopic and ultrastructural morphology of sarcocysts of *Sarcocystis* sp. in SD rats 5 months after inoculation with sporocysts isolated from rat snakes in Thailand. **a** Typical sporocyst from a fecal sample of *Coelognathus radiatus*; sporocysts from *C. flavolineatus* were identical in size and appearance; asterisk indicates single sporozoite. **b** Live sarcocyst, freed from muscle tissue; note the broad, palisade-like villar protrusions that could at times resemble those of *Sarcocystis singaporensis* with which this species can co-occur; however, the protrusions lack the basal stalks typical for the former species; the arrow highlights the septated compartments in the interior of the sarcocyst, and the inset shows a micrograph of live cystozoites freshly released from a cyst (arrowheads). **c** Typical structure of a cyst wall protrusion (isolate from *C. flavolineatus*); the arrows point to the electron-dense, knob-like structures of the primary cyst wall, whereby the knobs could apparently fuse to form an electron-dense borderline in larger protrusions (inset: arrowhead); also note the electron-light, thin layer of ground substance (GS) underneath the protrusions. **d** Typical cystozoites of the new species, which contained only two rhoptries (arrowheads) among relatively few micronemes (asterisks); additionally, the cystozoites exhibited vesicle-like structures in the anterior third of the cell containing electron-light, reticulate matter (arrow); the inset shows such a vesicle-like compartment at higher magnification, which was apparently not bound by a membrane (white arrow) and often located near micronemes (white asterisk); dense granules were present but rarely observed. **e** Interior and cyst wall of a mature sarcocyst (isolate from *C. radiatus*); metrocytes (asterisks) exclusively divided by endodyogeny, producing only two cystozoites (CZ). **f** Full-length section through a 15-µm-long protrusion of the sarcocyst wall; note that larger protrusions often occurred close to the tips of a cyst and showed a base with folds. AP, apicoplast; MI, mitochondrion; NU, nucleus; PT, villar protrusions

The sporocysts of *Sarcocystis* isolated from the two ratsnake species in Thailand, *Coelognathus radiatus* and *C. flavolineatus*, were indistinguishable in size, measuring on average 10.07 (± 0.39; s.d.) × 7.18 (± 0.25) µm (*n* = 20) and 10.28 (± 0.80) × 7.51 (± 0.73) µm (*n* = 21), respectively (Fig. 1a). After inoculation of six SD rats with both sporocyst isolates, we observed sarcocysts in the striated musculature of all six rats that were examined 5 months after infection. In fresh tissue preparations, sarcocysts of both isolates were numerous and morphologically identical, measuring 400–700 × 70–90 µm (length × diameter; *n* = 25 from four rats). We observed sarcocysts in the striated musculature of abdomen, thorax, limbs, diaphragm and esophagus; their form was cigar-shaped, while longer cysts could show slightly pointed ends. Live sarcocysts, which had been freed from host tissue, showed a rim of palisade-like, villar protrusions that were between 8 and 10 µm long and about 2–4 µm wide at their base; however, protrusions at the tip of a cyst could be longer, measuring between 10 and 16 µm with a base that was on average 3.6 µm wide (range: 2.5–4.1 µm, *n* = 15). The interior of native sarcocysts appeared to be septated (Fig. 1b). Banana-shaped cystozoites, freshly released from ruptured, native sarcocysts, were on average 5.9 (± 0.6; s.d.) µm long and 2.2 (± 0.4) µm in diameter (*n* = 21; both isolates).

Ultrastructurally, the mean diameter of the sarcocyst wall (= primary cyst wall [PCW] plus ground substance [GS]) was 10.16 (± 2.34) µm (*n* = 20), while the villar protrusions measured on average 8.65 (± 2.05; *n* = 20; two cysts, one each from each isolate) µm in length and 2.50 (± 0.61) µm in diameter. The electron-dense, knob-like structures of the PCW were about 150 nm wide at the base of the villar protrusions, whereas their width was about 75 nm in the more apical parts, where knobs could fuse to form a continuous layer of PCW (Fig. 1c). The layer of GS underneath the villar protrusions was thin, on average about 270 nm wide (Fig. 1c, e). Notably, cystozoites of the isolates from Thailand only possessed two rhoptries, which was evident from longitudinal as well as cross sections (Fig. 1d, e). In cross sections through the anterior third of cystozoites, we counted on average 60 micronemes (± 14; *n* = 6). These micronemes were loosely scattered throughout the anterior half of the cell; they appeared short and stumpy and were about 200 nm long. Dense granules were present but rarely observed. Metrocytes occurred regularly, more frequently towards the ends of a sarcocyst; apparently, they divided by endodyogeny since they contained only two zoites in all observed cases (Fig. 1e). As shown in Fig. 1f, large cyst wall protrusions could attain a length well over 10 µm.

The four negative control rats examined 5 months after infection were free of any *Sarcocystis* infection. All rats remained healthy throughout the experiment.

### Morphology of sarcocysts from wild rodents in Borneo and China

**Fig. 2:**
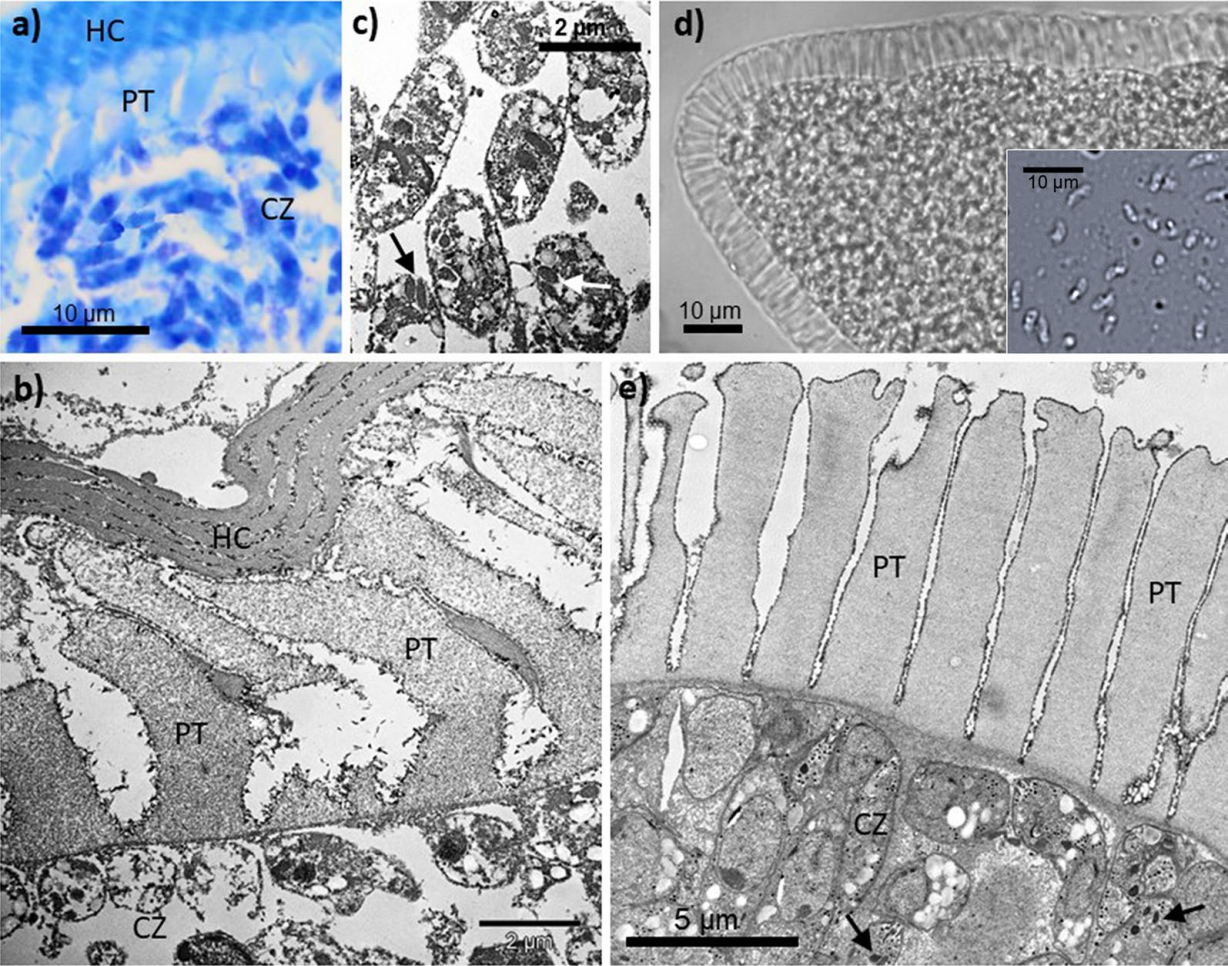
Light microscopic and ultrastructural morphology of sarcocysts from *Maxomys whiteheadi* in Borneo (**a**–**c**) and wild *Rattus norvegicus* in China (**d**, **e**). Note, due to ethanol fixation some ultrastructural details of the samples from Borneo are poorly resolved. **a** Richardsen’s dye-stained 1.0-µm thin section through a mature sarcocyst showing the villar protrusions (PT) of the cyst wall and numerous relatively small cystozoites (CZ). **b** Same sample as before under the electron microscope; note the thin layer of ground substance underneath the protrusions. **c** Enlarged part of the interior of the sarcocyst showing cystozoites—although with limited resolution—that possess a pair of rhoptries each, which is characteristic for this *Sarcocystis* species (black and white arrows; compare with Fig. 1d). **d** Live sarcocyst isolated from striated muscle tissue of a wild Norway rat in China; the inset shows live cystozoites that were freshly released from a cyst. **e** Ultrastructure of the same sarcocyst as before; note that the villar protrusions are highly similar to the samples from Borneo and Thailand regarding size and shape (Fig. 1e); again, cystozoites only exhibit one pair of rhoptries (arrows) and relatively few micronemes

Sarcocyst-infected muscle tissue from Borneo was isolated from two wild-caught *Maxomys whiteheadi* (Whitehead’s spiny rat), which showed numerous sarcocysts with a striated wall in histological sections of musculature of the abdominal wall. Because cysts were mainly in the cross-sectional plane, we can only provide their mean diameter, which was 85.6 (± 17.1) µm (*n* = 5). At higher magnification (Fig. 2a), the cyst wall consisted of broad, palisade-like protrusions which were on average 5.1 (± 2.5) µm long (*n* = 10). At the tip of one cyst, protrusions were longer, measuring 6.8–9.1 µm. However, because most of the protrusions in histological sections appeared to be bent or compressed, their actual size was probably larger, as measurements from live sarcocysts from other sampling locations suggested. The protrusions of the sarcocyst wall were indistinguishable from the samples from Thailand and China in terms of ultrastructure (Fig. 2b). Although preservation of ultrastructural details of the Bornean sample was relatively low, the typical, knobbed and electron-dense lining of the PCW of the protrusions was clearly visible including presence of electron-light granular material filling the core. On average, protrusions were 7005 (± 1235) nm long (*n* = 6); their diameter in the apical part ranged between 1940 and 3690 nm, at the base between 1625 and 2610 nm (*n* = 10); thus, some of the protrusions appeared enlarged towards the apical part. Two fully stretched cystozoites measured 4300–4940 × 1060–1259 nm, while all observed cystozoites possessed only two rhoptries like the isolates from Thailand (Fig. 2c). Cystozoites in H&E-stained histological sections measured 4.6 (± 0.4) × 1.2 (± 0.2) µm (*n* = 10).

Muscle cysts found in 14 out of 189 (7.4%) Norway rats from southwestern China were identical to those observed for the *Sarcocystis* isolates from Thailand and Borneo (Fig. 2e). There were numerous sarcocysts in the skeletal muscles, but none in the heart. Under the light microscope, live sarcocysts measured 770–1580 × 87–123 μm in size (*n* = 20). The cysts had palisade-like villar protrusions measuring 8.5–11 μm in length and 1.7–2.4 μm in width (*n* = 30) (Fig. 2d); they were filled with numerous banana-shape cystozoites measuring 6.5 (± 0.6; s.d.) × 2.1 (± 0.15) μm in size (*n* = 25). At the ultrastructural level, the primary cyst wall contained numerous vertical, palisade-like protrusions, measuring 6.5–10.8 × 1.6–3.1 μm in size (*n* = 30), which contained electron-light granular matter in their core (Fig. 2e). The PCW consisted of a knobbed, electron-dense layer and showed minute undulations over its entire surface. A layer of ground substances with 200–300 nm thickness was located immediately underneath the primary sarcocyst wall.

### Morphological comparison of the novel *Sarcocystis *sp. with closely related taxa

**Fig. 3:**
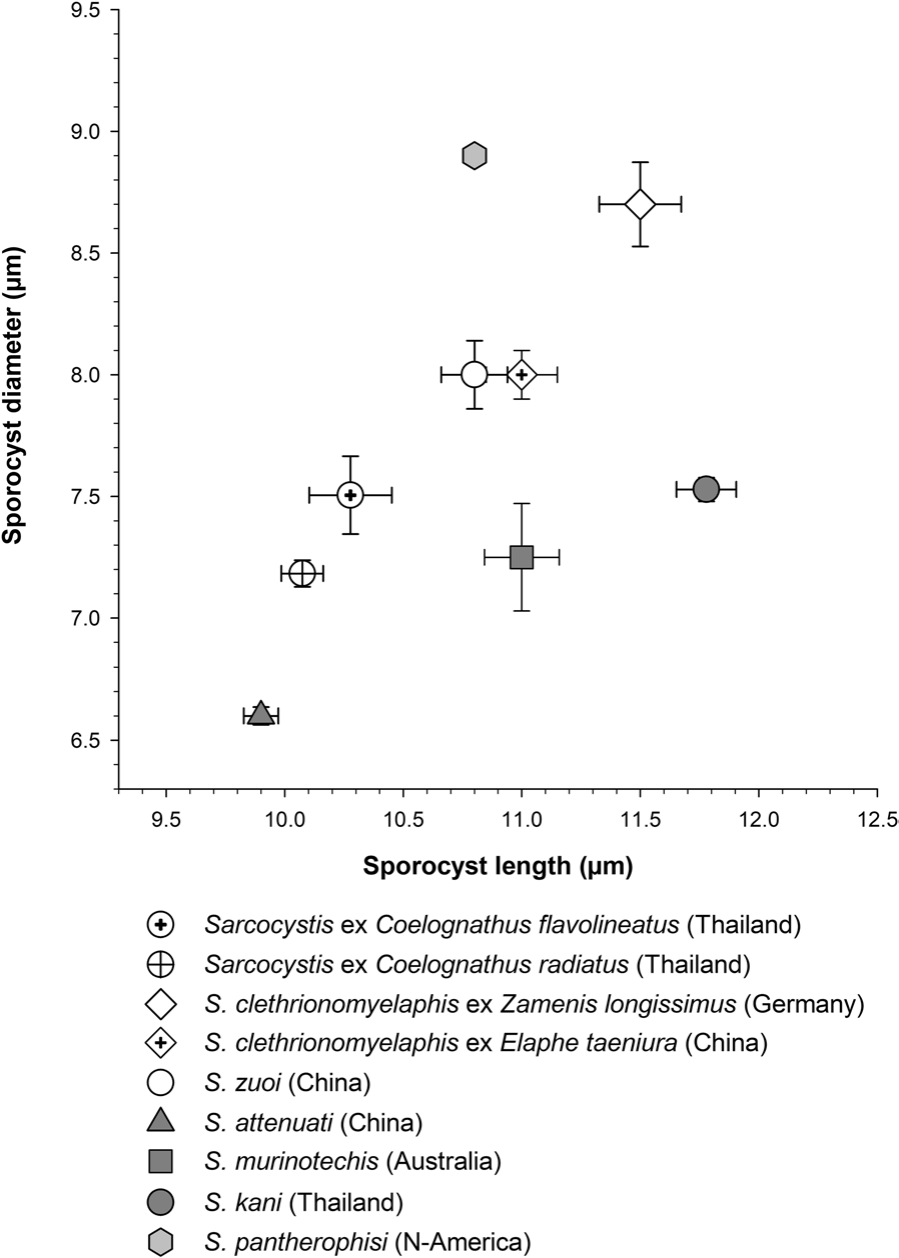
Graph showing the size of sporocysts (length plotted against diameter, in μm; error bars indicate s.e.) of the *Sarcocystis* isolates from the colubrid snakes *Coelognathus flavolineatus* and *C. radiatus* in Thailand and closely related *Sarcocystis*. Every isolate/species is indicated by a different symbol (legend), whereby sporocyst samples with the same shape index (= length/diameter) share the same background shading: white = 1.3; dark = 1.5; *Sarcocystis* *pantherophisi* = 1.2. Here, *S. pantherophisi* is included as reference for the snake host *Sarcocystis* lineage S2, while all other species belong to lineage S1 (except for *S*. *murinotechis*, for which no genetic information is available)

The mean (± s.d.) sporocyst length and diameter of both isolates of *Sarcocystis* sp. from Thailand given as 10.07 (± 0.39) × 7.18 (± 0.25) µm (*n* = 20) (from *Coelognathus radiatus*) and 10.28 (± 0.80) × 7.51 (± 0.73) µm (*n* = 21) (from *C*. *flavolineatus*) compared to other studies as follows: *Sarcocystis clethrionomyelaphis* isolated in Germany (11.5 [± 0.6] × 8.7 [± 0.6] μm, *n* = 12; [36]), *S. clethrionomyelaphis* from China (11.0 [± 0.8] × 8.0 [± 0.5] μm, *n* = 25; [37]), *S. zuoi* from China (10.8 [± 0.7] × 8.0 [± 0.7] μm, *n* = 25; [11]), *S. attenuati* isolated in China (9.9 [± 0.4] × 6.6 [± 0.2] μm, *n* = 30; [23]), *S*. *kani* from Thailand (11.8 [± 0.5] × 7.5 [± 0.2] μm, *n* = 14; [6]) and *S. murinotechis* from Australia (11.0 [± 0.5] × 7.25 [± 0.7] μm, *n* = 10; [7]) (Fig. 3). Sporocyst lengths of *Sarcocystis* sp. from *C. radiatus* were significantly shorter than in *S. clethrionomyelaphis*, *S*. *kani*, *S. murinotechis* and *S*. *zuoi* (Holm-Sidak post hoc* P* < 0.001 following one-way ANOVA test with *F*_(7, 149)_ = 22.5, *P* < 0.001) but not in the case of *S. attenuati* (*P* = 0.321) and *Sarcocystis* sp. isolated from *C. flavolineatus* (*P* = 0.489). The mean sporocyst diameter of *Sarcocystis* sp. from *C*. *radiatus* was significantly larger than that measured for *S*. *attenuati* and smaller than for* S*. *clethrionomyelaphis*, *S*. *kani* and *S*. *zuoi* (post hoc* P* < 0.001 following ANOVA with *F*_(7, 149)_ = 30.4, *P* < 0.001) whereas no apparent difference was found in the measured diameters of *S. murinotechis* (*P* = 0.738) and *Sarcocystis* sp. isolated from *C. flavolineatus* (*P* = 0.136); the power of all performed tests with alpha = 0.05 was 1.

We also examined the size of live cystozoites of the new *Sarcocystis* sp. from Thailand and China (mean length × diameter in µm and s.d.: Thailand, 5.9 [± 0.6] × 2.2 [± 0.4], *n* = 21; China, 6.5 [± 0.6] × 2.1 [± 0.15], *n* = 25) in comparison with *S. clethrionomyelaphis* from China (9.5 [± 1.0] × 2.7 [± 0.1], *n* = 40; same authors as above), *S*. *kani* (7.7 [± 0.4] × 2.5 [± 0.2], *n* = 7), *S. zuoi* (8.5 [± 0.8] × 2.8 [± 0.1], *n* = 35), *S. attenuati* (9.2 [± 0.7] × 2.5 [± 0.3], *n* = 40) and *S. eothenomysi* (6.8 [± 0.4] × 3.1 [± 0.3], *n* = 23; [38]); the latter species represented lineage S2. In comparison, the isolate of *Sarcocystis* sp. from Thailand was indistinguishable in size from the isolate of *Sarcocystis* sp. from China but was significantly different from all other examined species: The cystozoite length of *Sarcocystis* sp. from Thailand was significantly shorter than those of all studied species (post hoc Holm-Sidak test *P* < 0.05 with ANOVA *F*_(6, 170)_ = 80.565, *P* < 0.001) but indistinguishable in length from those in *R*. *norvegicus* in China (*P* = 0.074). Likewise, cystozoite diameters of *Sarcocystis* sp. from Thailand were significantly smaller than those from all other species (post hoc* P* < 0.05 with ANOVA *F*_(6, 170)_ = 50.6, *P* ≤ 0.001) but indistinguishable in size from those in *R*. *norvegicus* from China (*P* = 0.187); power of all performed tests = 1 (alpha = 0.05).

Because there were no data on the size of cystozoites of *S. murinotechis* available, we re-examined the length of its cystozoites taken from an image of a sarcocyst in muscle tissue published by Munday et al. [18] that was associated with *S. murinotechis* [7]. In this sarcocyst, cystozoites of *S. murinotechis* were 7.2 (± 0.8) µm long (*n* = 12). In our own histological sections of sarcocysts of *Sarcocystis* sp. from rats inoculated with sporocysts from *Coelognathus radiatus* and *C*. *flavolineatus*, cystozoites measured 4.2 (± 0.5) µm (*n* = 14) and 4.5 (± 0.9) µm (*n* = 6) in length, respectively. When we compared these samples of *Sarcocystis* sp. (plus cystozoite length in tissue sections from Borneo, see previous section) with data on *S*. *kani* [6], *S. scandentiborneensis* [20] and the measurements of *S. murinotechis*, cystozoites of *Sarcocystis* sp. from Thailand (ex *C*. *radiatus*) and Borneo (ex *M*. *whiteheadi*) were significantly shorter than *S*. *kani*, *S. murinotechis* and *S. scandentiborneensis* (Holm-Sidak’s *P* < 0.001 with ANOVA *F*_(5, 136)_ = 35.3, *P* < 0.001) but not different in intraspecific comparison: *Sarcocystis* sp. ex *C. radiatus* versus *Sarcocystis* sp. ex *M. whiteheadi* from Borneo*; P* = 0.198; *Sarcocystis* sp. ex *C*. *radiatus* versus *Sarcocystis* sp. ex *C*. *flavolineatus*; *P* = 0.360; power of all performed tests = 1 (alpha = 0.05).

Taken together, these comparisons provided substantial evidence that the new species showed significant morphological disparity (including its sporocysts) to *S. murinotechis*, *S. zuoi* and other *Sarcocystis* spp., while the cystozoites of *Sarcocystis* sp. from Thailand, Borneo and China were similar in size.

### Molecular evidence for the new *Sarcocystis* sp. from intermediate hosts in Borneo and China and other novel *Sarcocystis* sequences from forest rodents

In China, targeted genetic markers were successfully sequenced from four individuals of *R*. *norvegicus*, including the nuclear *18S* and *28S rRNA* genes, the ITS1-*5.8S*-ITS2 *rRNA* gene complex and mitochondrial *cox1* (Additional file 1: Table S1). From the samples in Borneo, we were able to amplify three partial *28S rRNA* sequences from cyst-infected musculature of two specimens of *M. whiteheadi*, of which the sequence E369.13-28S (GenBank OR979643) stood out because of its reasonable length (610 nt) useful for phylogenetic analysis. The other two sequences from a second specimen of *M. whiteheadi* were too short for tree building (isolates E389.13A-28S, 142 bp, and E389.13B-28S, 137 bp) but 100% identical with isolate E369.13-28S (Additional file 1: Table S1), potentially indicating that these sequences also belonged to the new *Sarcocystis* sp. Moreover, sarcocyst morphology (LM and TEM) in the second forest rat was identical to that in the first specimen (Fig. 2b, c). We also amplified a sequence from another forest-dwelling rat, *Niviventer cremoriventer* (OR979644, isolate E388.13A-28S, 413 nt), which was 99.03% identical to the sequences of *Sarcocystis* sp. from Borneo and China but showed the highest identity score (99.76%) with sequence E357.13-KLP1F (OR979647; Additional file 1: Table S1), a partial *28S rRNA* sequence that was amplified from the same sarcocyst DNA (isolated from a treeshrew) which was deposited as holotype of *Sarcocystis scandentiborneensis* [20]. Although *S. scandentiborneensis* was so far known to develop in treeshrews only, we amplified two more putative *28S rRNA* gene sequences of this species from muscle samples of rodents: here, two specimens of Low’s squirrel *Sundasciurus lowii*, while these sequences (E346.13-28S, E367.13-28S) showed the highest identity score (99.82%) with E357.13-KLP1F of *S. scandentiborneensis* in pairwise comparisons (Additional file 1: Table S1). The light microscopic appearance and ultrastructure of the corresponding sarcocysts from the squirrels also corresponded to features of *S. scandentiborneensis*, corroborating the link to that species. Presumably intraspecific sequence comparisons at around 600-bp alignment length of the *Sarcocystis* sp. *28S rRNA* E369.13-28S from *M. whiteheadi* with isolate Rn-cl2.3-28S from Norway rats in China (including the other haplotypes) and KU341120 from *R. tiomanicus* in Thailand all resulted in 99% identity. Although sequence *S. scandentiborneensis* E357.13-KLP1F also showed 99% identity with E369.13-28S of the new *Sarcocystis* sp. from Whitehead’s spiny rat, the alignment length under pairwise BLAST analysis was around 60 bp shorter, apparently too short to include the bp differences that would have separated the two species by the similarity score. This was indicated by the result that another (putative) sequence of *S*. *scandentiborneensis* that was similar in length (614 bp, isolate E367-13-28S, OR979645) only showed a similarity score of 98.37% in pairwise alignment with sequence E369.13-28S; both sequences included the expansion segment D2 at the 3’-end. Moreover, character-based phylogenetic reconstruction of the *28S rRNA* tree under the ML criterion using the same sequences could distinguish well between the two species (see below). Importantly, BLAST comparisons of *Sarcocystis* sp. from Borneo and China with a newly sequenced, full-length *28S rRNA* of *S*. *zuoi* (OR979648, isolate RN-cl2-3-28S) revealed a sequence identity of only 97% indicating that *Sarcocystis* sp. was different from *S*. *zuoi* at the *28S rRNA* locus (Additional file 1: Table S1).

Since we could not examine our own DNA samples of *Sarcocystis* sp. from Thailand, we regarded isolate KU341120 from Thailand as the most likely candidate to be conspecific with the former species. Apart from the high identity scores between the *28S rRNA* sequence KU341120 and the partial, homologous sequences of *Sarcocystis* sp. from Borneo and China (in the latter comparison over a stretch of about 1500 bp), alignment of the *18S rRNA* gene sequence KU341120 with the new, full-length *18S rRNA* from China (*Sarcocystis* sp. haplotypes Zhils1-18S and Zhils2-18S) resulted in identity scores > 99% over a length of 1700 alignment positions (Additional file 1: Table S1). This included the highly variable domains V2 and V4 according to the predicted secondary structure of the *T. gondii* reference sequence [27]. Taken together, these results strongly suggested that the *28S* and *18S rRNA* gene sequences deposited under KU341120 represented DNA of *Sarcocystis* sp. from Thailand. In contrast to the results on the *28S rRNA* gene mentioned above, *Sarcocystis* sp. *18S rRNA* haplotypes Zhils1-18S and Zhils2-18S showed the highest identity scores with the type sequences of *S*. *zuoi* (JQ029112, JQ029113), which made the two taxa indistinguishable at the *18S rRNA* locus (Additional file 1: Table S1). However, one must consider here that the *S*. *zuoi* sequences were truncated [20] so that a potential divergence between the taxa may have been concealed.

We observed the greatest sequence variability in the ITS1*-5.8S-*ITS2 gene complex, whereby the most relevant (and available) sequences for comparison with *Sarcocystis* sp. from China (isolates Cl312-ITS/5.8S, Cl41-ITS/5.8S, Cl43-ITS/5.8S, Cl45-ITS/5.8S) were KU341120 and those from *S. attenuati*. Sequence identity of *Sarcocystis* sp. with KU341120 ranged between 92 and 93% (1268–1276 alignment positions), whereas this value was 89% for comparisons with various haplotypes of *S. attenuati* over 712–736 alignment positions (Additional file 1: Table S1). Although we had amplified various sequences of the ITS complex from *S*. *zuoi* in China, the amplicons only included ITS1 and *5.8S rRNA* and are therefore not listed in Additional file 1: Table S1. However, we used the excised ITS1 of *S*. *zuoi* for phylogenetic analysis (below).

Four new sequences of the *cox1* gene of *Sarcocystis* sp. (only one sequence deposited: PP033596, isolate 11526FR1-Cox1) were not only completely identical among each other but also highly similar to *S*. *kani*, *S. scandentiborneensis*, *S. attenuati* and *S*. *zuoi* (Additional file 1: Table S1): The new *Sarcocystis* sp. differed from *S*. *kani* in only three out of 927 homologous positions, in five out of 1333 bp compared to *S. attenuati* (MZ889673), in three out of 976 bp in the case of *S. scandentiborneensis* and in eight out of 978 bp compared to the newly sequenced *cox1* gene of *S*. *zuoi* (PP033597, isolate SF1-Rm1011-Cox1). Its divergence compared to a recently discovered *Sarcocystis* from *Rattus argentiventer* in Sumatra (*Sarcocystis* sp. 2, ON989200) was higher with 20 out of 986 bp changes. Since all these taxa are considered part of the molecularly cryptic *S*. *zuoi* complex of species [6], the new results here show that the latter two species exhibited the greatest divergence at the *cox1* locus within this group, although overall variability was relatively low. Furthermore, it confirmed for *cox1* (as for the *28S rRNA*) that *Sarcocystis* sp. isolated from Norway rats in China was different from the original *S. zuoi* isolated from the same intermediate host in China. Sequence divergence of *Sarcocystis* sp. compared to *S*. *singaporensis* and *S*. *zamani* from pythonid hosts was higher (Additional file 1: Table S1). The latter two species and the *S*. *zuoi*—complex are included in the so-called S1-lineage of snakehost *Sarcocystis* spp. in the *18S rRNA* phylogenetic tree [35], whereby the *cox1* gene tree topology is similar to the *18S rRNA* except that members of *S*. *zuoi* complex form a polytomic clade [6]. The same polytomy also resulted after phylogenetic analysis of *cox1* of the new *Sarcocystis* species, which is why we do not show the *cox1* tree here. The new *cox1* sequences contained the barcode area, which in the case of *Sarcocystis* sp. started with a complete, in the case of *S. zuoi* incomplete helix 1, as exemplified for *S. scandentiborneensis* [20].

### Phylogenies of the *28S, 18S rRNA* genes and ITS1 region

**Fig. 4:**
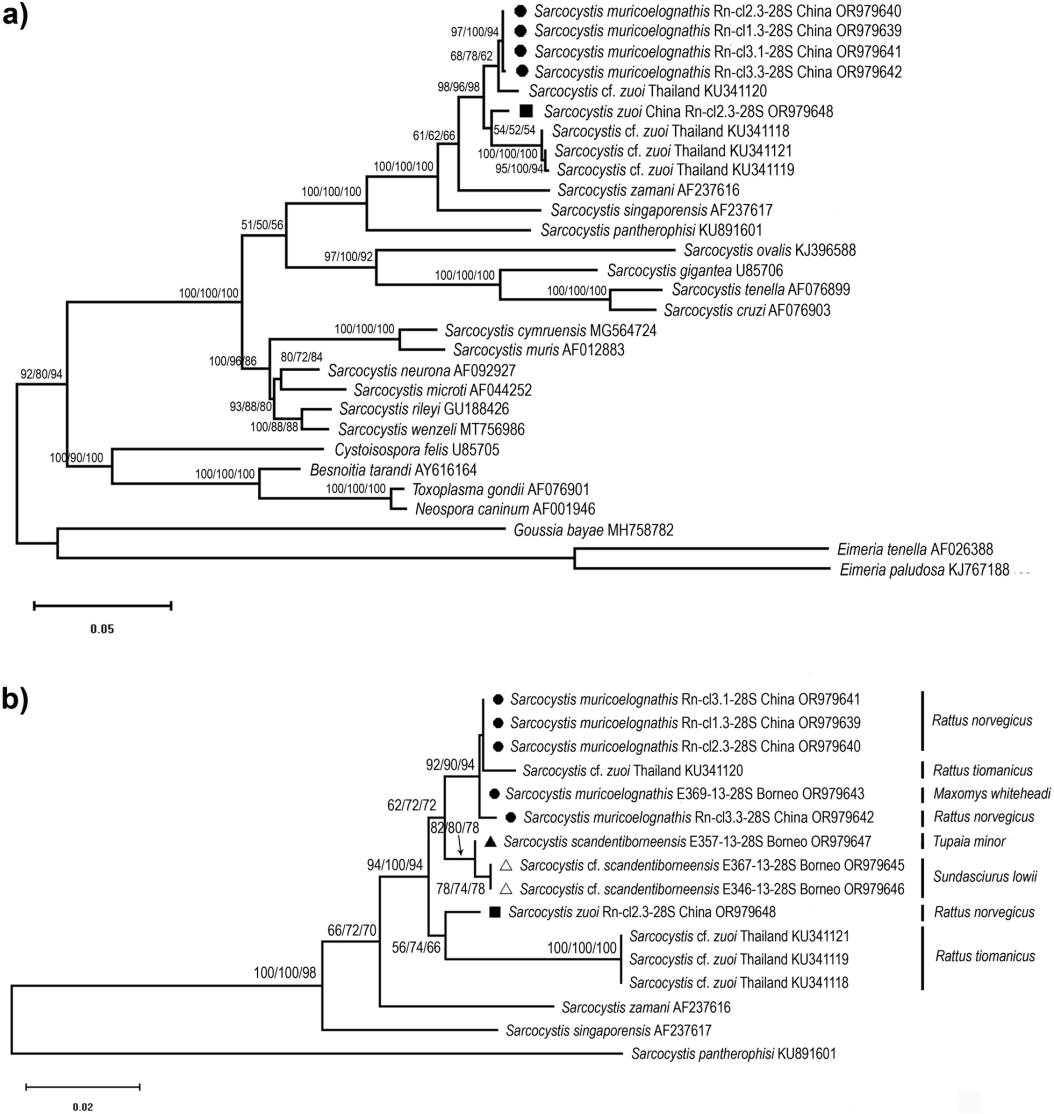
Two separate phylogenies of the *28S rRNA* gene (longer and shorter sequence fragments) of the new *Sarcocystis* sp. sampled in China, newly sequenced *S*. *zuoi* from China and novel *Sarcocystis* isolates from Borneo. Symbols indicate the new sequences of this study, whereby taxa considered conspecific are grouped by shape. GenBank accession numbers are given behind each taxon name. **a** Maximum likelihood (ML) analysis of an alignment of 29 sequences and 1383 homologous positions. Branch support by bootstrapping (1000 replicate trees) is shown next to the branches, whereby the results of three independent analyses based on independent alignments are shown. The scale bar indicates the number of substitutions per site. All positions with < 85% site coverage were eliminated, i.e. fewer than 15% alignment gaps, missing data and ambiguous bases were allowed at any position (partial deletion option). Selected eimeriid coccidia served as outgroup. **b** ML analysis of a trimmed alignment including five shorter sequences of *Sarcocystis* sampled in Borneo compared with the samples of *Sarcocystis* sp. from China; a total of 16 sequences and 366 homologous positions with site coverage of 95% were compared. *Sarcocystis pantherophisi* served as outgroup. The corresponding natural intermediate hosts are also indicated

Phylogenetic reconstruction of the *28S rRNA* gene tree was performed considering longer sequences (comparing about 1400 homologous positions) as well as shorter sequences to accommodate the samples from Borneo (400–500 sites). Maximum likelihood (ML) analysis with 29 longer sequences, including *Sarcocystis* sp. from Norway rats in China, sequence KU341120 isolated from *R*. *tiomanicus* in Thailand and *S*. *zuoi* from *R. norvegicus* in China revealed that the former two taxa clustered together while *S*. *zuoi* was a sister species included in a separate clade that also contained the sequences KU341118/19/21 from *R*. *tiomanicus* in Thailand (Fig. 4a). The *Sarcocystis* species from pythonid snakes (*S*. *singaporensis*, *S*. *zamani*) branched off basally from the monophyletic clade shared with the *Sarcocystis* spp. of interest. *Sarcocystis pantherophisi*, the only representative of the S2 lineage for this marker, branched off separately from the S1 cluster with high branch support. Subsequently, we used *S*. *pantherophisi* as outgroup in a smaller tree of 16 *28S rRNA* sequences that included the shorter amplicons of the samples from Borneo. As shown in Fig. 4b, all sequences of *Sarcocystis* sp. from China and KU341120 from Thailand formed a monophyletic clade that also included isolate E369-13-28S from *M. whiteheadi* in Borneo. Hence, all three geographic locations of the novel species were combined in one monophyletic clade with relatively high branch support, which was congruent with the high identity scores observed in the BLAST analyses (Additional file 1: Table S1). A sister clade contained the two sequences retrieved from the squirrel *Sundasciurus lowii* that clustered with *S*. *scandentiborneensis* (isolate E357.13-28S). Similar to the topology of the *28S rRNA* tree based on longer sequences, *S*. *zuoi* clustered with sequences KU341118/19/21, apart from the new *Sarcocystis* species.

**Fig. 5:**
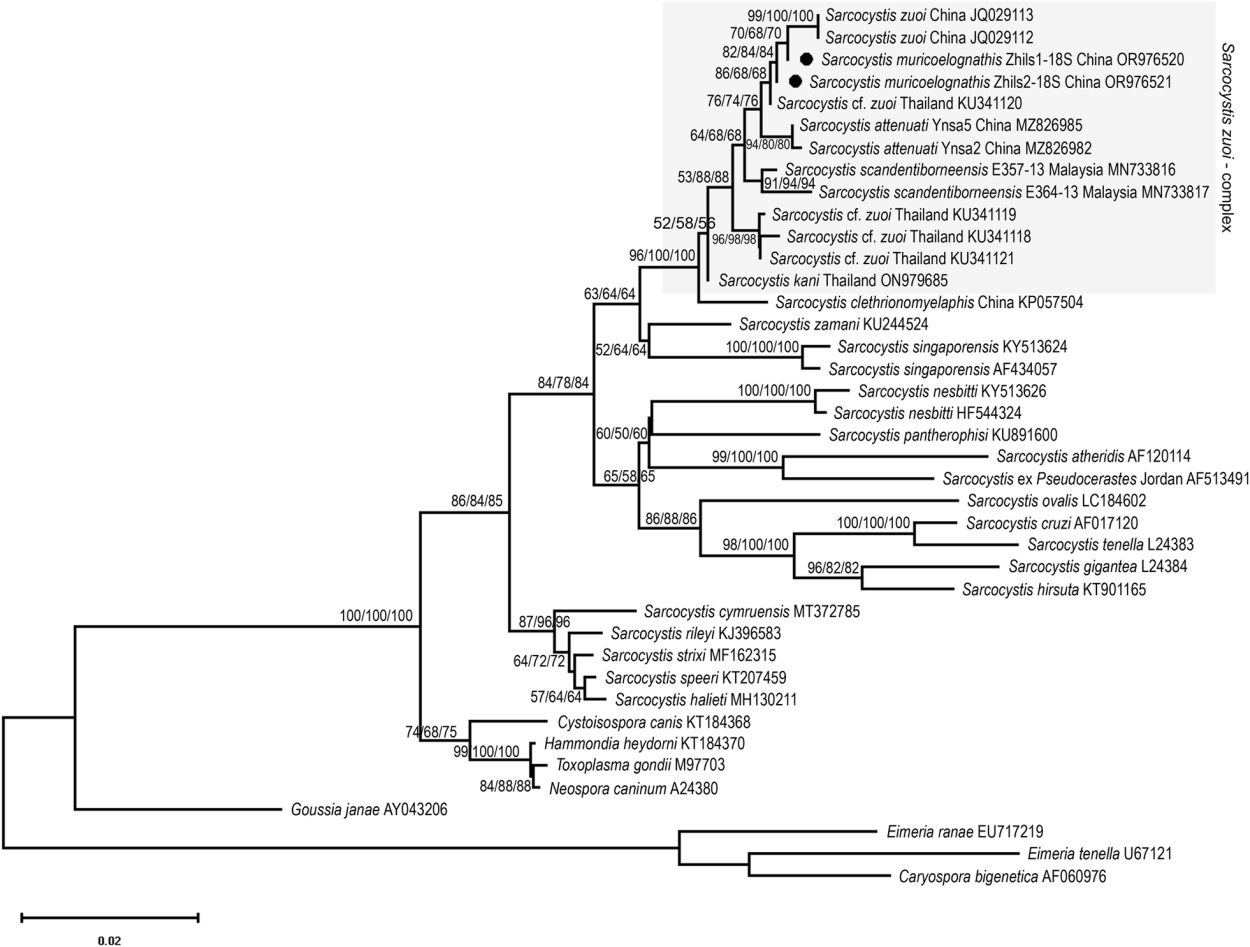
Phylogenetic reconstruction of the *18S rRNA* gene tree of the novel *Sarcocystis* species and other tissue cyst-forming coccidia based on 1465 homologous positions of 40 aligned nucleotide sequences under the minimum evolution (ME) criterion; selected eimeriid coccidia served as outgroup. The new sequences of *Sarcocystis* sp. from China are highlighted by black symbols. Branch support values are shown for 1000 bootstrap replicates of three independent alignments with a site coverage of 95%. The shaded box highlights the taxa included in the so-called *S. zuoi* complex

A different tree topology resulted after reconstruction of the *18S* r*RNA* tree (albeit after inclusion of additional members of the *S*. *zuoi* complex, such as *S*. *attenuati* and *S*. *kani*), where *Sarcocystis* sp. and sequence KU341120 clustered with *S*. *zuoi* in a fashion that could not unequivocally distinguish between these taxa: Character-based ML analysis produced a polytomic clade with very short branch lengths, while the distance-based ME algorithm resulted in a dichotomic topology of this clade which suggested that isolate KU341120 and *Sarcocystis* sp. assumed an ancestral position relative to *S*. *zuoi* (Fig. 5): There was no speciation time/branching (sensu [39]) of *Sarcocystis* sp. after its split from *S. zuoi*. Similar to the *28S rRNA* tree, the clade containing the members of the *S*. *zuoi* complex showed good bootstrap support and species of the S2 snakehost lineage (e.g. *S*. *pantherophisi*, *Sarcocystis* *nesbitti*, *S*. *atheridis*) were part of a separate cluster of sequences.

**Fig. 6:**
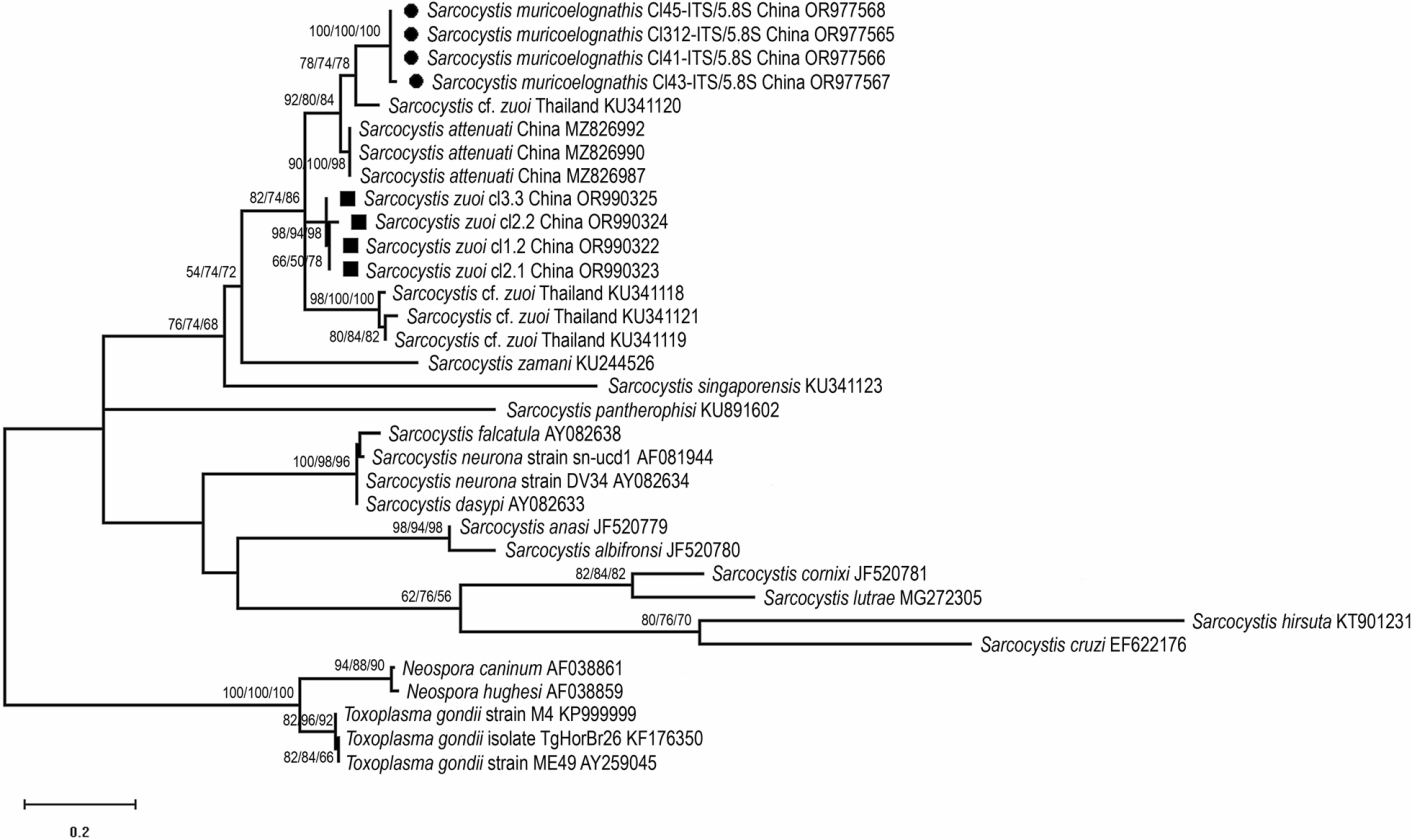
ML analysis of the ITS1 region of *Sarcocystis* sp., *S. zuoi* and other species of the Sarcocystidae; members of the Toxoplasmatinae served as outgroup. Bootstrap branch support values are shown in triplicate, indicating results from three independent alignments and analyses. The tree is drawn to scale, with branch lengths measured in the number of substitutions per site. The analysis involved 204 homologous positions of 33 nucleotide sequences

Since the 18S *rRNA* tree could not unequivocally distinguish between *S*. *muricoelognathis* and *S*. *zuoi*, and the *cox1* locus was largely uninformative regarding the *Sarcocystis* species of interest (Additional file 1: Table S1), we also reconstructed the phylogeny of the ITS1 locus (after excision from the ITS1*-5.8S-*ITS2 complex deposited under OR977565-68). We hoped to find additional evidence for the divergence between *Sarcocystis* sp. and *S*. *zuoi*, which apparently use the same intermediate hosts in the wild in China. Although alignment of the ITS1 sequences was demanding because of their high variability (i.e. relatively few conserved positions shared between species), secondary structure-guided alignment and subsequent ML analysis resulted in a tree topology that was well in agreement with the *28S rRNA* tree: The ITS1 sequences of *Sarcocystis* sp. and *Sarcocystis* isolate KU341120 clustered together, while the newly sequenced ITS1 of *S*. *zuoi* (OR990322-25) formed a separate clade with high branch support (Fig. 6). Similarly, *S*. *singaporensis* and *S*. *zamani* branched off basally from the shared S1 clade, and *S*. *pantherophisi* was located on a separate branch.

### Taxonomic summary of *Sarcocystis muricoelognathis* sp. nov.

(*Sarcocystis* sp.: Figs. 1, 2, 3, 4, 5, 6, Additional file 1: Table S1).

#### Diagnosis

Oocyst containing two sporocysts with four sporozoites each; sporocysts measuring on average 10.1–10.3 × 7.2–7.5 µm; sarcocysts microscopic with striated cyst wall in fresh preparations (similar to *S. singaporensis* but cyst wall protrusions not resting on stalks), cigar-shaped with slightly pointed ends in older cysts; in skeletal musculature except heart; native sarcocysts in rats infected experimentally for 5 months measured 400–700 × 70–90 µm, in wild-caught rats 770–1580 × 87–123 μm; cyst wall with palisade-like, villar protrusions, 8 to 11 µm long and 2–4 µm wide at their base; protrusions at the tips of the sarcocyst often longer, 10–16 µm; banana-shaped, live cystozoites on average 5.9–6.5 µm long and 2.1–2.2 µm in diameter; ultrastructurally, villar protrusions palisade-like, attached upright to the cyst (if not bent by preparation), sometimes slightly broadened towards the apical ends, delimited by a knob-like lining and filled with fine, electron-light granular material; cystozoites with only two rhoptries throughout distribution range.

#### Type natural definitive hosts

*Coelognathus radiatus* (holotype), *Coelognathus flavolineatus* (paratype).

#### Type experimental intermediate host

*Rattus norvegicus* (holotype)

#### Type natural intermediate hosts

*Maxomys whiteheadi* (paratype); *Rattus norvegicus* (paratype)

#### Type localities

Holotype: central Bangkok, Thailand; paratypes: Chumphon, southern Thailand; Poring Hot Springs, Northern Borneo, Sabah, Malaysia; Anning and Nanjian Prefectures, China.

#### Other locations and natural hosts not included in type series

Sarcocysts with the same ultrastructure recorded from *Rattus argentiventer* and *Bandicota indica* in Thailand [2] and reported from *Rattus rattus* in Singapore [15].

#### Etymology

Species name in reference to the intermediate and definitive hosts.

#### Type specimens deposited

Holotype deposited at the Department of Parasitology, University of Hohenheim, Emil-Wolff-Str. 34, 70599 Stuttgart-Hohenheim, Germany, accession number DP/R41C961764: Resin-embedded sarcocyst from *Rattus norvegicus* infected with sporocysts from *Coelognathus radiatus*; paratype from Borneo deposited at the Sabah Parks Museum, Kinabalu Park, Sabah, Malaysia, accession number SP/EAC/00102: sarcocyst in musculature from *Maxomys whiteheadi*; paratypes from China deposited at the Zoological Specimen Museum of Yunnan University, Kunming, accession number Prot202302: formalin-fixed tissue containing sarcocysts as well as photomicrographs.

#### Sequences deposited at GenBank

*18S rRNA*: OR976520, OR976521; *28S rRNA*: isolates from China OR979639, OR979640, OR979641, OR979642, and Borneo OR979643; ITS1*-5.8S-*ITS2: OR977565, OR977566, OR977567, OR977568; *cox1*: PP033596.

#### ZooBank Registration

This article has been submitted to ZooBank under the Life Science Identifier (LSID) urn:lsid:zoobank.org:pub:E86A1FEF-6189-4E28-A627-11ABFAA635AC. The LSID for the new species name *Sarcocystis muricoelognathis* is urn:lsid:zoobank.org:act:000AF647-D4AC-4609-914D-61C1449A2DAA.

## Discussion

### Taxonomic remarks

Since the ultrastructure of the sarcocyst wall is in many cases a useful criterion for distinguishing between species of *Sarcocystis* [40], we show here that *S. muricoelognathis* is clearly distinct from two similar species, *S*. *zuoi* and *S*. *murinotechis*. In the case of *S*. *zuoi*, its villar protrusions of the cyst wall are bent by a certain angle, which is also visible in live sarcocysts when the surrounding host tissue has been removed, and exhibit a reticulate base [11]. In contrast, *S. muricoelognathis* shows upright protrusions with a largely non-reticulate base. Compared to *S. murinotechis* from Australia [7], the two former species have markedly longer protrusions (roughly twice as long). Although only two electron micrographs of the sarcocysts of *S*. *murinotechis* exist in the original species description, the accompanying text characterizes them as possessing “short, broad protrusions” that were about 6 µm in length [7]. Relatively short protrusions are also visible in a histological section of a sarcocyst of *S*. cf. *murinotechis* from *Rattus lutreolus* [18] supporting the observations on *S*. *murinotechis* published by these authors 2 years later. All in all, we propose that the sarcocyst ultrastructure of *S. muricoelognathis* could be categorized under group 10a of the common cyst wall classification system for *Sarcocystis* [40]. Additionally, the sizes of the transmission stages, sporocysts and cystozoites, were significantly different among the three species discussed above. Notably, sporocysts of *S*. *murinotechis* exhibited a shape index different from *S*. *muricoelognathis* and *S*. *zuoi*: Size differences of sporocysts can be regarded a suitable indicator for the potential presence of different *Sarcocystis* species [41]. Regarding intraspecific variability, cystozoites of *S*. *muricoelognathis* were remarkably constant in size independent of geographic location and intermediate host and consistently showed two rhoptries at the ultrastructural level. These characteristics underscore the uniqueness of *S. muricoelognathis* compared to other species with striated cyst walls, which we have discussed previously [20]. At the light microscopic level, sarcocysts of *S*. *muricoelognathis* may be easily confused with *S*. *singaporensis*, because both species are microscopic, show a striated cyst wall with relatively long protrusions, exhibit pointed sarcocyst ends and share similar geographic distributions and rodent hosts. However, the cyst wall protrusions of the latter species rest on typical stalks, which appear as a thin, basal layer of the striated wall at lower magnification in live and histological preparations [2]. Molecularly, *S*. *muricoelognathis* can be unequivocally identified using its *28S rRNA* and ITS1 sequences, whereby the partial *28S rRNA* gene (≥ 600 bp) is already sufficient for differential diagnosis if the variable expansion segments D1 and D2 are included (as discussed below).

In a taxonomic context, we think we provided substantial molecular evidence for the proposition that the rRNA sequences from Thailand deposited under accession number KU341120 [19] do not belong to *S. zuoi* but represent *S. muricoelognathis*. Although a high degree of sequence identity between partial sequences alone may be insufficient for species discrimination, we must add here that the *28S rRNA* gene sequence of *S. muricoelognathis* from Borneo included the variable expansion segments D1 and D2 of the rRNA molecule according to the predicted secondary structure of the reference sequence of *T. gondii* (L25635, [27]). Especially, domain D2 has been shown to be highly variable [42]. Moreover, the overlapping parts of the alignments of partial sequence KU341120 with the (almost) full-length *28S rRNA* sequences of *S. muricoelognathis* from China included expansion segments D1–D6, which, in view of an identity score of 99%, strongly suggested that these sequences belong to the same species. Furthermore, both taxa clustered together in the ITS1 phylogenetic tree, which is increasingly used for discriminating between closely related *Sarcocystis* spp. (e.g. [43]) and shows much greater evolutionary rates than other rRNA markers [28]. Given that the sequences under KU341120 have been included in major rRNA databases under the species name ‘*Sarcocystis zuoi*’ and are frequently cited in the context of *Sarcocystis* of rodents (e.g. [44, 45]), it will probably take some time until any annotation in line with our proposal could be expected. Moreover, based on the observations here and a previous study [6], it can be safely assumed that the sequences KU341118/19/21 also do not represent *S*. *zuoi* but belong to a different species, because they clustered in a clade apart from the original *S*. *zuoi* sequences.

### Close phylogenetic relationships between *S. muricoelognathis* and its sister species

*Sarcocystis muricoelognathis* is a new member of what we call the *S. zuoi* complex, a group of *Sarcocystis* species cycling between colubrid snakes and small mammals in eastern Asia that shows remarkably close genetic relationships but significant morphological disparity regarding sarcocyst ultrastructure as well as the sizes of its transmission stages [6]. Close genetic relatedness was especially apparent among *S. muricoelognathis*, *S. attenuati* [23], *S*. *kani* [6] and *S. scandentiborneensis* [20], from which the former differed in ≤ 5-bp changes within the partial mitochondrial *cox1* gene. The *cox1* gene sequences included the barcode fragment, which is used globally for discrimination between species of the tree of life [46]. Although the newly sequenced *cox1* gene of *S. zuoi*, the indicator species of this complex, showed a few additional bp changes, its identity score also reached 99% compared to the species above. This suggests incomplete lineage sorting (ILS) at this locus and a recent common ancestor of these taxa. Mitochondrial DNA shows a relatively rapid rate of mutation, which makes it suitable as a marker for more recent evolutionary history of natural populations [47, 48]. In contrast, the *28S rRNA* and ITS1 trees could distinguish well between *S. muricoelognathis* and its sister species, which was indicated by a mostly dichotomic tree resolution and absence of ILS, which can be recognized by polyphyly or paraphyly in fast evolving species [49, 50]. Although the *18S rRNA* gene was also useful for discriminating between various taxa of the *S*. *zuoi* complex, it could not unequivocally resolve the divergence between *S*. *zuoi* and *S*. *muricoelognathis* in this study for the reason outlined earlier. In the future, a full-length *18S rRNA* of *S*. *zuoi* should be sequenced to aid in the further study of this group of *Sarcocystis* spp. The fast-evolving ITS1 marker (e.g. compare scale bars of Figs. 5 and 6) produced results congruent with the *28S* and *18S rRNA* gene trees. We can confirm that the alignment of ITS sequences was challenging because of their high variability and often incorrect sequence boundaries in public databases [51]. We are confident that careful determination of starting and end points of ITS1 and using a secondary structure-guided sequence alignment (because ITS folds into stems, bulges and loops; [52]) helped find the correct tree.

The close genetic relatedness among the species of the *S. zuoi* complex as apparent in the *cox1* phylogeny [6] and revealed here for *S. muricoelognathis* as well was also reflected in the relatively short branch lengths of this clade in the *18S rRNA* gene tree. This contrasted with longer branch lengths of the species of the S2 snakehost lineage (sensu [35]), such as *S. pantherophisi*, *S. atheridis* or *S. nesbitti*. Differences in branch lengths can indicate variable evolutionary rates among the coccidia [34] but could also be a result of different evolutionary histories [53]. In view of this, we hypothesized that the *Sarcocystis* species of the *S*. *zuoi* complex may undergo radiation that followed the documented recent radiations of their intermediate and definitive hosts in Asia [6].

### Host shifting of *Sarcocystis* between commensal and forest rodents in the biodiversity hotspot Borneo

Remarkably, *S. muricoelognathis* exhibits a relatively wide distribution range in Asia (China, Thailand, northern Borneo and probably also Singapore) and apparently utilizes forest and commensal rodents as intermediate hosts. A wide distribution range is in line with other *Sarcocystis* species from colubrid snakes, notably, with *S. clethrionomyelaphis*, which was originally isolated in Germany from *Zamenis longissimus* [36] and later in China from *Elaphe taeniura* [37]. Also, the geographical range of *S. zuoi* was previously already assumed to include China, Thailand, Malaysia and Japan [11, 19, 54, 55], respectively. However, we show here that the four *18S rRNA* gene sequences from Thailand previously designated as ‘*S. zuoi*’ are not conspecific with *S*. *zuoi* as discussed above. Furthermore, other sequences of the same gene with the same species designation collected in Malaysia and Japan may not be *S. zuoi*, because they were relatively short and not accompanied by a morphological description of a sarcocyst [54, 55]. Hence, based on current evidence *S. zuoi* records are from China only; however, that does not exclude the possibility that this species has a wider distribution than currently anticipated.

The island of Borneo with its dipterocarp tropical forests is a global biodiversity hotspot for mammalian species [56]. In a previous study, we showed that *S. scandentiborneensis* is a parasite infecting treeshrews in Borneo [20]. Here, we found first molecular and morphological indications that the intermediate host range of *S. scandentiborneensis* includes the rat *Niviventer cremoriventer* and the squirrel *Sundasciurus lowii*. Although these findings require further confirmation, especially in the case of *Niviventer*, they hint at the possibility that the intermediate host range of this species in its forest habitat might be larger than previously anticipated. Our observation that *S. muricoelognathis* occurs in commensal and forest rodents points in the same direction; we observed the new *Sarcocystis* species in the rat *Maxomys whiteheadi*, which is frequently found in primary and logged forest in Borneo but exhibits limited habitat overlap with commensal *Rattus* spp. [57]. While *S. muricoelognathis* is a prevalent encounter in commensal rats in China (this study), Thailand [2] and on Singaporean islands [15], the newly reported *Sarcocystis* species with snake-rodent life cycle in forest habitats question the origin of associations with rodents and the possible history of host shifting events. In this context, the study of Paperna et al. [15] is interesting insofar as they showed that infection rates with *S. singaporensis* of the commensal rodent *Rattus rattus* were higher in forest habitats on islands that were less disturbed by human activity, i.e. that were more likely to harbor the pythonid definitive host at densities that could maintain the parasite’s life cycle. A follow-up study in Singapore about 12 years later confirmed the trend that parasite transmission was negatively affected by expanding urbanization [58]. Furthermore, a recent phylogenetic study on the evolution of dietary preferences of colubrid snakes found that *Coelognathus flavolineatus* and *C. radiatus* are prototypes of a dietary generalist that occupied tropical forests in Asia [59]. The evolutionary history of rat snakes traces back to the forests of Southeast Asia, where their ancestors originated around 37 million years ago [60, 61]. Moreover, although *Sarcocystis singaporensis* is one of the most prevalent and widely distributed coccidian species in commensal rats of the Indo-Australian Archipelago [35], its intermediate host spectrum on Java and Sulawesi in Indonesia appears to be greatly extended to a variety of forest rodents [10].

This growing body of insight suggests that transmission cycles between snakes and rodents are closed in both forest and anthropogenically modified habitats, questioning whether commensal rodents or those confined to natural forest habitats were the original intermediate hosts. In this context, it was hypothesized that infection of phylogenetically older intermediate hosts like shrews and treeshrews by phylogenetically younger species of the *S*. *zuoi* complex (i.e. *S*. *attenuati* and *S*. *scandentiborneensis*, respectively) might be the result of host shifting or adaptive processes rather than co-speciation [6]. Our finding that *S*. *scandentiborneensis* also likely occurs in sciurid rodents highlights the possibility that such a host shift could have occurred from the Rodentia to the Scandentia. For eimeriid coccidia infecting rodents, the occurrence of repeated host switches among closely related genetic lineages has been established [62]. Furthermore, the paradigm of *Sarcocystis* spp. being highly host-specific for their intermediate hosts might require a more differentiated analysis, since deviations from this generalization have been observed for *Sarcocystis* spp. infecting wild ruminants and farm animals [63, 64], members of the lineage clustering with *Sarcocystis neurona* (e.g. *S*. *canis*, [43]) and, early on, *Sarcocystis* spp. with snake-rodent life cycle [65].

## Conclusions

*Sarcocystis muricoelognathis* sp. nov. cycles between colubrid snakes as definitive and forest and commensal rodents as intermediate hosts, further increasing the number of species of the so-called *S*. *zuoi* complex, members of which appear to have expanded intermediate host ranges. Although we anticipate that these host associations are likely only snapshots of the true associations, our results provide evidence of broad geographic distributions of rodent-associated *Sarcocystis* and host shifts between commensal and forest small mammals. Future work is required to better understand the possible eco-evolutionary pathways of host shifting of *Sarcocystis* species, which also deserves attention from a contemporary conservation and disease control perspective.

### Supplementary Information

**Additional file 1: Table S1.** Pairwise sequence comparisons using the novel sequences of *Sarcocystis* sp., *S. scandentiborneensis* and *S. zuoi* and their association with sarcocyst morphology where possible (remarks).

## Data Availability

All relevant data of this study are contained within the manuscript, while type materials of the new *Sarcocystis* species have been deposited in the institutions as indicated in the taxonomic summary section. All new DNA sequences are publicly available at GenBank under the following accession numbers: ***18S rRNA***: *Sarcocystis* sp. isolates Zhils1-18S, Zhils2-18S (OR976520, OR976521); ***28S rRNA***: *Sarcocystis* sp. isolates Rn-cl1.3-28S, Rn-cl2.3-28S, Rn-cl3.1-28S, Rn-cl3.3-28S (OR979639, OR979640, OR979641, OR979642); *Sarcocystis* sp. isolate E369-13-28S (OR979643); *Sarcocystis* cf. *scandentiborneensis* isolates E388-13A-28S, E367-13-28S, E346-13-28S (OR979644, OR979645, OR979646); *Sarcocystis scandentiborneensis* isolate E357-13-28S (OR979647); *Sarcocystis zuoi* isolate Rn-cl2.3-28S (OR979648); **ITS1-*****5.8S rRNA-*****ITS2**: *Sarcocystis* sp. isolates Cl312-ITS-5.8S, Cl41-ITS-5.8S, Cl43-ITS-5.8S, Cl45-ITS-5.8S (OR977565, OR977566, OR977567, OR977568); **ITS1**: *Sarcocystis zuoi* isolates cl1.2, cl2.1, cl2.2, cl3.3 (OR990322, OR990323, OR990324, OR990325); ***cox1***: *Sarcocystis* sp. isolate 11526FR1-Cox1 (PP033596); *Sarcocystis zuoi* isolate SF1-Rm1011-Cox1 (PP033597).

## Abbreviations

ANOVA: Analysis of variance
*cox1*: Mitochondrial Cytochrome oxidase subunit 1 gene
ES: Eukaryotic expansion segments of the rRNA
ITS: Internal transcribed spacer region
ME: Minimum evolution criterion
ML: Maximum likelihood criterion
PCR: Polymerase chain reaction
s.d.: Standard deviation
s.e.: Standard error of the mean
SD rats: Sprague-Dawley rats
*18S*, * 5.8S* and, *28**S**rRNA*: Nuclear 18S, 5.8S and 28S ribosomal RNA genes

## References

[1] Ashford RW (1978). *Sarcocystis cymruensis* n. sp., a parasite of rats *Rattus norvegicus* and cats *Felis catus*. Ann Trop Med Parasitol.

[2] Jäkel T, Khoprasert Y, Sorger I, Kliemt D, Seehabutr V, Suasa-ard K (1997). Sarcosporidiasis in rodents from Thailand. J Wildl Dis.

[3] Hu JJ, Liao JY, Meng Y, Guo YM, Chen XW, Zuo YX (2011). Identification of *Sarcocystis cymruensis* in wild *Rattus flavipectus* and *Rattus norvegicus* from Peoples Republic of China and its transmission to rats and cats. J Parasitol.

[4] Antunes Murata FH, Cerqueira-Cezar CK, Thompson PC, Tiwari K, Mowery JD, Verma SK (2018). *Sarcocystis cymruensis*: discovery in Western Hemisphere in the Brown rat (*Rattus norvegicus*) from Grenada, West Indies: redescription, molecular characterization, and transmission to IFN-gamma gene knockout mice via sporocysts from experimentally infected domestic cat (*Felis catus*). Parasitol Res.

[5] Zeng H, Guo Y, Ma C, Deng S, Hu J, Zhang Y (2020). Redescription and molecular characterization of sarcocysts of *Sarcocystis cymruensis* from Norway rats (*Rattus norvegicus*) and *Sarcocystis ratti* from black rats (*R. rattus*) in China. Parasitol Res.

[6] Jäkel T, Raisch L, Richter S, Wirth M, Birenbaum D, Ginting S (2023). Morphological and molecular phylogenetic characterization of *Sarcocystis kani* sp. nov. and other novel, closely related *Sarcocystis* spp. infecting small mammals and colubrid snakes in Asia. Int J Parasitol Parasites Wildl.

[7] Munday BL, Mason RW (1980). Sarcocystis and related organisms in Australian wildlife: III.
*
Sarcocystis murinotechis
*
sp.n. life cycle in rats (
*
Rattus
*
,
*
Pseudomys
*
and
*
Mastocomys
*
spp.) and tiger snakes (
*
Notechis ater
*
). J Wildl Dis.

[8] Prakas P, Kirillova V, Gavarane I, Gravele E, Butkauskas D, Rudaityte-Lukosiene E (2019). Morphological and molecular description of *Sarcocystis ratti* n. sp. from the black rat (*Rattus rattus*) in Latvia. Parasitol Res.

[9] Beaver PC, Maleckar JR (1981). *Sarcocystis singaporensis* Zaman and Colley, (1975) 1976, *Sarcocystis villivillosi* sp. n., and *Sarcocystis zamani* sp. n.: development, morphology, and persistence in the laboratory rat, *Rattus norvegicus*. J Parasitol.

[10] O’Donoghue PJ, Watts CH, Dixon BR (1987). Ultrastructure of *Sarcocystis* spp. (Protozoa: Apicomplexa) in rodents from North Sulawesi and West Java, Indonesia. J Wildl Dis.

[11] Hu JJ, Meng Y, Guo YM, Liao JY, Song JL (2012). Completion of the life cycle of *Sarcocystis zuoi*, a parasite from the Norway rat, *Rattus norvegicus*. J Parasitol.

[12] Munday BL (1983). An isosporan parasite of masked owls producing sarcocysts in rats. J Wildl Dis.

[13] Grikieniene J, Arnastauskiene T, Kutkiene L (1993). On some disregarded ways of sarcosporidians circulation and remarks about systematics of the genus *Sarcocystis* Lankester, 1882 with the description of the new species from rodents. Ekologija.

[14] Slapeta JR, Modry D, Votypka J, Jirku M, Koudela B, Lukes J (2001). Multiple origin of the dihomoxenous life cycle in sarcosporidia. Int J Parasitol.

[15] Paperna I, Peh KSH, Martelli P, Koh LP, Sodhi NS (2004). Factors affecting *Sarcocystis* infection of rats on small tropical islands. Ecol Res.

[16] Lee FCH (2019). Finding *Sarcocystis* spp. on the Tioman Island: 28S rRNA gene next-generation sequencing reveals nine new *Sarcocystis* species. J Water Health.

[17] Uetz P, Freed P, Aguilar R, Reyes F, Hošek J. The Reptile Database. http://www.reptile-database.org. 2022.

[18] Munday BL, Mason RW, Hartley WJ, Presidente PJ, Obendorf D (1978). *Sarcocystis* and related organisms in Australian wildlife: I. Survey findings in mammals. J Wildl Dis.

[19] Watthanakaiwan V, Sukmak M, Hamarit K, Kaolim N, Wajjwalku W, Muangkram Y (2017). Molecular characterization of the ribosomal DNA unit of *Sarcocystis singaporensis*, *Sarcocystis zamani* and *Sarcocystis zuoi* from rodents in Thailand. J Vet Med Sci.

[20] Ortega Pérez P, Wibbelt G, Brinkmann A, Galindo Puentes JA, Tuh FYY, Lakim MB, Nitsche A (2020). Description of *Sarcocystis scandentiborneensis* sp. nov. from treeshrews (*Tupaia minor*, *T. tana*) in northern Borneo with annotations on the utility of COI and 18S rDNA sequences for species delineation. Int J Parasitol Parasites Wildl.

[21] Aplin KP, Brown PR, Jacob J, Krebs CJ, Singleton GR (2003). Field methods for rodent studies in Asia and the Indo-Pacific.

[22] Musser G, Carleton M, Wilson DE, Reeder DM (2005). Superfamily muroidea. Mammal species of the world: a taxonomic and geographic reference.

[23] Hu J, Sun J, Guo Y, Zeng H, Zhang Y, Tao J (2022). Infection of the Asian gray shrew *Crocidura attenuata* (Insectivora: Soricidae) with *Sarcocystis attenuati* n. sp. (Apicomplexa: Sarcocystidae) in China. Parasit Vectors.

[24] Jäkel T (1995). Cyclic transmission of *Sarcocystis gerbilliechis* n. sp. by the Arabian saw-scaled viper, *Echis coloratus*, to rodents of the subfamily Gerbillinae. J Parasitol.

[25] Prakas P, Oksanen A, Butkauskas D, Sruoga A, Kutkiene L, Svazas S (2014). Identification and intraspecific genetic diversity of
*
Sarcocystis rileyi
*
from ducks, Anas spp., Lithuania and Finland. J Parasitol.

[26] Tamura K, Stecher G, Peterson D, Filipski A, Kumar S (2013). MEGA6: molecular evolutionary genetics analysis version 6.0. Mol Biol Evol.

[27] Gagnon S, Bourbeau D, Levesque RC (1996). Secondary structures and features of the 18S, 5.8S and 26S ribosomal RNAs from the *Apicomplexan* parasite *Toxoplasma gondii*. Gene.

[28] Bass D, Richards TA, Matthai L, Marsh V, Cavalier-Smith T (2007). DNA evidence for global dispersal and probable endemicity of protozoa. BMC Evol Biol.

[29] Clark K, Karsch-Mizrachi I, Lipman DJ, Ostell J, Sayers EW (2017). GenBank. Nucleic Acids Res.

[30] Tommaso PD, Moretti S, Xenarios I, Orobitg M, Montanyola A, Chang JM (2011). T-Coffee: a web server for the multiple sequence alignment of protein and RNA sequences using structural information and homology extension. Nucleic Acids Res.

[31] Kumar S, Stecher G, Li M, Knyaz C, Tamura K (2018). MEGA X: molecular evolutionary genetics analysis across computing platforms. Mol Biol Evol.

[32] Rzhetsky A, Nei M (1992). A simple method for estimating and testing minimum evolution trees. Mol Biol Evol.

[33] Tamura K, Kumar S (2002). Evolutionary distance estimation under heterogeneous substitution pattern among lineages. Mol Biol Evol.

[34] Morrison DA, Bornstein S, Thebo P, Wernery U, Kinne J, Mattsson JG (2004). The current status of the small subunit rRNA phylogeny of the coccidia (Sporozoa). Int J Parasitol.

[35] Wassermann M, Raisch L, Lyons JA, Natusch DJD, Richter S, Wirth M (2017). Examination of *Sarcocystis* spp. of giant snakes from Australia and Southeast Asia confirms presence of a known pathogen—*Sarcocystis nesbitti*. PLoS ONE.

[36] Matuschka F-R (1986). *Sarcocystis clethrionomyelaphis* n. sp. from snakes of the genus *Elaphe* and different voles of the family *Arvicolidae*. J Parasitol.

[37] Hu JJ, Liu TT, Liu Q, Esch GW, Chen JQ (2015). *Sarcocystis clethrionomyelaphis* Matuschka, 1986 (Apicomplexa: Sarcocystidae) infecting the large oriental vole *Eothenomys miletus* (Thomas) (Cricetidae: Microtinae) and its phylogenetic relationships with other species of *Sarcocystis* Lankester, 1882. Syst Parasitol.

[38] Hu JJ, Liu Q, Yang YF, Esch GW, Guo YM, Zou FC (2014). *Sarcocystis eothenomysi* n. sp. (Apicomplexa: Sarcocystidae) from the large oriental vole *Eothenomys miletus* (Thomas) (Cricetidae: Microtinae) from Anning, China. Syst Parasitol.

[39] Hedges SB, Marin J, Suleski M, Paymer M, Kumar S (2015). Tree of life reveals clock-like speciation and diversification. Mol Biol Evol.

[40] Dubey JP, Calero-Bernal R, Rosenthal BM, Speer CA, Fayer R (2016). *Sarcocystosis* of animals and humans.

[41] McAllister CT, Upton SJ, Trauth SE, Dixon JR (1995). Coccidian parasites (Apicomplexa) from snakes in the southcentral and southwestern United States: new host and geographic records. J Parasitol.

[42] Mugridge NB, Morrison DA, Jäkel T, Heckeroth AR, Tenter AM, Johnson AM (2000). Effects of sequence alignment and structural domains of ribosomal DNA on phylogeny reconstruction for the protozoan family *Sarcocystidae*. Mol Biol Evol.

[43] Leger JS, Chen Y, Sakamaki K, Mena A, Raverty SA, Rotstein D (2023). Fatal hepatic sarcocystosis in three captive and one free-ranging pinniped. Int J Parasitol Parasites Wildl.

[44] Canova V, Helman E, Robles MDR, Abba AM, More G (2023). First report of *Sarcocystis* spp. (Apicomplexa, Sarcocystidae) in *Lagostomus maximus* (Desmarest, 1917) (Rodentia, Chinchillidae) in Argentina. Int J Parasitol Parasites Wildl.

[45] Prakas P, Stirke V, Sneideris D, Rakauskaite P, Butkauskas D, Balciauskas L (2023). Protozoan parasites of *Sarcocystis* spp. in rodents from commercial Orchards. Animals.

[46] Pentinsaari M, Salmela H, Mutanen M, Roslin T (2016). Molecular evolution of a widely-adopted taxonomic marker (COI) across the animal tree of life. Sci Rep.

[47] Brown WM, George M, Wilson AC (1979). Rapid evolution of animal mitochondrial DNA. Proc Natl Acad Sci USA.

[48] Norman JA, Blackmore CJ, Rourke M, Christidis L (2014). Effects of mitochondrial DNA rate variation on reconstruction of Pleistocene demographic history in a social avian species, *Pomatostomus superciliosus*. PLoS ONE.

[49] Belfiore NM, Liu L, Moritz C (2008). Multilocus phylogenetics of a rapid radiation in the genus *Thomomys* (Rodentia: Geomyidae). Syst Biol.

[50] Maddison WP, Knowles LL (2006). Inferring phylogeny despite incomplete lineage sorting. Syst Biol.

[51] Bengtsson-Palme J, Ryberg M, Hartmann M, Branco S, Wang Z, Godhe A (2013). Improved software detection and extraction of ITS1 and ITS2 from ribosomal ITS sequences of fungi and other eukaryotes for analysis of environmental sequencing data. Methods Ecol Evol.

[52] Rampersad SN (2014). ITS1, 5.8S and ITS2 secondary structure modelling for intra-specific differentiation among species of the *Colletotrichum gloeosporioides* sensu lato species complex. SpringerPlus.

[53] Molnar K, Ostoros G, Dunams-Morel D, Rosenthal BM (2012). *Eimeria* that infect fish are diverse and are related to, but distinct from, those that infect terrestrial vertebrates. Infect Genet Evol.

[54] Lau YL, Chang PY, Subramaniam V, Ng YH, Mahmud R, Ahmad AF (2013). Genetic assemblage of *Sarcocystis* spp. In Malaysian snakes. Parasit Vectors.

[55] Abe N, Matsubara K, Tamukai K, Miwa Y, Takami K (2015). Molecular evidence of *Sarcocystis* species in captive snakes in Japan. Parasitol Res.

[56] Struebig MJ, Wilting A, Metcalfe K, Kramer-Schadt S (2015). Targeted conservation to safeguard a biodiversity hotspot from climate and land-cover change. Curr Biol.

[57] Wells K, Kalko EK, Lakim MB, Pfeiffer M (2007). Effects of rain forest logging on species richness and assemblage composition of small mammals in Southeast Asia. J Biogeogr.

[58] Devan-Song A, Luz S, Mathew A, Low MR, Bickford DP (2017). Pythons, parasites, and pests: anthropogenic impacts on *Sarcocystis* (Sarcocystidae) transmission in a multi-host system. Biotropica.

[59] Barends JM, Maritz B (2022). Dietary specialization and habitat shifts in a clade of Afro-Asian colubrid snakes (Colubridae: Colubrinae). Ichthyol Herpetol.

[60] Burbrink FT, Lawson R (2007). How and when did old world ratsnakes disperse into the new world?. Mol Phylogenet Evol.

[61] Rage J-C, Seigel Richard A, Collins Joseph T, Novak Susan S (1987). Fossil record. Snakes: ecology and evolutionary biology.

[62] Macova A, Hoblikova A, Hypsa V, Stanko M, Martinu J, Kvicerova J (2018). Mysteries of host switching: diversification and host specificity in rodent-coccidia associations. Mol Phylogenet Evol.

[63] Dahlgren SS, Gjerde B (2010). Molecular characterization of five *Sarcocystis* species in red deer (*Cervus elaphus*), including *Sarcocystis hjorti* n. sp., reveals that these species are not intermediate host specific. Parasitology.

[64] Marandykina-Prakiene A, Butkauskas D, Gudiskis N, Juozaityte-Ngugu E, Bagdonaite DL, Kirjusina M (2013). *Sarcocystis* species richness in sheep and goats from Lithuania. Vet Sci.

[65] Matuschka FR (1987). Reptiles as intermediate and/or final hosts of *Sarcosporidia*. Parasitol Res.

[66] Barta JR, Martin DS, Liberator PA, Dashkevicz M, Anderson JW, Feighner SD (1997). Phylogenetic relationships among eight *Eimeria* species infecting domestic fowl inferred using complete small subunit ribosomal DNA sequences. J Parasitol.

[67] Fischer S, Odening K (1998). Characterization of bovine *Sarcocystis* species by analysis of their 18S ribosomal DNA sequences. J Parasitol.

[68] Fenger CK, Granstrom DE, Langemeier JL, Stamper S, Donahue JM, Patterson JS (1995). Identification of opossums (*Didelphis virginiana*) as the putative definitive host of *Sarcocystis neurona*. J Parasitol.

[69] Mugridge NB, Morrison DA, Johnson AM, Luton K, Dubey JP, Votypka J (1999). Phylogenetic relationships of the genus *Frenkelia*: a review of its history and new knowledge gained from comparison of large subunit ribosomal ribonucleic acid gene sequences. Int J Parasitol.

