# Supplementary material for: A cyst-forming coccidian with large geographical range infecting forest and commensal rodents: Sarcocystis muricoelognathis sp. nov
Source: Parasit Vectors. 2024 Mar 15;17:135. doi: 10.1186/s13071-024-06230-8 (PMC10943850; doi:10.1186/s13071-024-06230-8)
Supplement: Supplementary file 1 — Additional file 1: Table S1. Pairwise sequence comparisons using the novel sequences of Sarcocystis sp., S. scandentiborneensis and S. zuoi and their association with sarcocyst morphology where possible (remarks). [file 13071_2024_6230_MOESM1_ESM.docx]

**Table S1** Pairwise sequence comparisons using the novel sequences of *Sarcocystis* sp., *S. scandentiborneensis*, and *S. zuoi* and their association with sarcocyst morphology where possible (remarks).

| Gene | Novel sequence accession number (species/isolate) | Length (bp) | Intermediate host (geography) | Intraspecific haplotype identity (%) | Pairwise sequence comparisons (% identity and gaps) | Remarks |
| --- | --- | --- | --- | --- | --- | --- |
| *18S rRNA* | OR976520 (*Sarcocystis* sp., Zhils1-18S) | 1859 | *Rattus norvegicus* (China) | 99.9 – 100 | *versus* KU341120, identities:1690/1701 (99.35%), gaps: 3/1701 (0%) | Corresponding sarcocyst identified (Fig 2d, e) |
|  |  |  |  |  | *versus* *S*. *zuoi*, JQ029113, identities: 1331/1332 (99.92%), gaps: 0/1332 (%) |  |
|  |  |  |  |  | *versus* *S*. *zuoi*, JQ029112, identities: 1347/1348 (99.93%), gaps: 0/1348 (0%) |  |
|  |  |  |  |  | *versus* *S. kani*, ON979685, identities: 1200/1216 (98.68%), gaps: 8/1216 (0%) |  |
|  |  |  |  |  | *versus* *Sarcocystis* sp.2 (Sumatra), ON979684, identities: 1685/1729 (97.46%), gaps: 17/1729 (0%) |  |
|  |  |  |  |  | *versus* *S. attenuati*, MZ826981, identities: 1840/1872 (98.29%), gaps: 20/1872 (1 %) |  |
|  |  |  |  |  | *versus* *S. scandentiborneensis*, MN733816; identities: 1802/1830 (98.47 %), gaps: 12/1830 (0%) |  |
|  |  |  |  |  | *versus* KU341119, identities: 1721/1759 (97.84 %), gaps: 10/1759 (0%); |  |
|  | OR976521 (*Sarcocystis* sp. Zhils2-18S) | 1859 | *Rattus norvegicus* (China) | 99.9 – 100 | *versus* KU341120: identities: 1691/1701 (99.41%), gaps: 3/1701 (0%) | Sarcocyst identified (Fig 2d, e) |
|  |  |  |  |  | *versus* *S*. *zuoi*, JQ029113, identities: 1332/1332 (100%), gaps: 0/1332 (0%) |  |
|  |  |  |  |  | *versus* *S*. *zuoi*, JQ029112, identities: 1348/1348 (100%), gaps: 0/1348 (0%) |  |
|  |  |  |  |  | *versus* *S. kani*, ON979685, identities: 1201/1216 (98.77%), gaps: 8/1216 (0%) |  |
|  |  |  |  |  | *versus* *Sarcocystis* sp.2 (Sumatra), ON979684, identities: 1686/1729 (97.51%), gaps: 17/1729 (0%) |  |
|  |  |  |  |  | *versus* KU341118: identities: 1704/1744 (97.71%), gaps: 10/1744 (0%) |  |
|  |  |  |  |  | *versus* *S. attenuati* MZ826985: identities: 1838/1872 (98.18%), gaps: 17/1872 (0%) |  |
|  |  |  |  |  | *versus* *S. scandentiborneensis* MN733816: identities: 1803/1830 (98.52%), gaps: 12/1830 (0%) |  |
| *28S rRNA* | OR979639  (*Sarcocystis* sp. Rn-cl1.3-28S) | 3364 | *Rattus norvegicus* (China) | 99.5 – 99.7 | *versus* KU341120: identities:1503/1520 (98.88%), gaps:9/1520 (0%) | Sarcocyst identified (Fig 2d, e); alignment with KU341120 and other, shorter sequences included ES D1 to D6^a^ |
|  |  |  |  |  | *versus* KU341121: identities: 871/922 (94.47%), gaps: 19/922 (2%) |  |
|  |  |  |  |  | *versus* *S. zuoi* (see below) |  |
|  | OR979640 (*Sarcocystis* sp. Rn-cl2.3-28S) | 3370 | *Rattus norvegicus* (China) | 99.5 – 99.7 | *versus* KU341120: identities:1503/1520 (98.88%), gaps: 9/1520 (0%) | Sarcocyst identified (Fig 2d, e); alignment with KU341120 and other, shorter sequences included ES D1 to D6 |
|  |  |  |  |  | *versus* KU341118: identities:766/799 (95.87%), gaps: 10/799 (1%); |  |
|  |  |  |  |  | *versus* *S. zuoi* (see below) |  |
|  | OR979641 (*Sarcocystis* sp. Rn-cl3.1-28S) | 3365 | *Rattus norvegicus* (China) | 99.5 – 99.7 | *versus* KU341120: identities:1500/1520 (98.68%), gaps: 8/1520 (0%) | Sarcocyst identified (Fig 2d, e); alignment with KU341120 and other, shorter sequences included ES D1 to D6 |
|  |  |  |  |  | *versus* KU341119: identities: 839/888 (94.48%), gaps: 18/888 (2%) |  |
|  |  |  |  |  | *versus* *S*. *zuoi* (see below) |  |
|  | OR979642 (*Sarcocystis* sp. Rn_cl3.3-28S) | 3365 | *Rattus norvegicus* (China) | 99.5 – 99.7 | *versus* KU341120: identities: 1502/1521 (98.75%), gaps: 10/1521 (0%) | Sarcocyst identified (Fig 2d, e); alignment with KU341120 included ES D1-D6 |
|  |  |  |  |  | *versus* *S*. *zuoi* (see below) |  |
|  | OR979643  (*Sarcocystis* sp. E369-13-28S) | 610 | *Maxomys whiteheadi* (Borneo) | n.a. | *versus Sarcocystis* sp*.* Rn-cl1.3-28S, identities: 607/614 (98.86%), gaps: 4/614 (0%) | Sarcocyst identified (Fig 2a, b); partial sequence, includes variable ES D1 and D2, and regions 10, 11, 12, 13, 14, 15, and 16 |
|  |  |  |  |  | *versus* Rn-cl2.3/3.1/3.3-28S: all 98.86% |  |
|  |  |  |  |  | *versus* KU341120**:** identities: 607/614 (98.86%), gaps: 4/614 (0%) |  |
|  |  |  |  |  | *versus* KU341121: identities: 591/610 (96.89%), gaps: 2/610 (0%) |  |
|  |  |  |  |  | *versus* KU341119: identities: 591/611 (96.73%), gaps: 2/611 (0%) |  |
|  |  |  |  |  | *versus* *S*. *scandentiborneensis* E357-13-28S, identities: 542/547 (99.09%), gaps 0/547 (0%) |  |
|  |  |  |  |  | *versus S*. cf. *scandentiborneensis* E367-13-28S, identities: 604/614 (98.37%), gaps: 4/614 (0%) |  |
|  |  |  |  |  | *versus S. zuoi* (see below) |  |
|  |  |  |  |  | *versus* *S*. *pantherophisi* KU891601: identities: 523/592 (88.34%), gaps:25/592 (4 %) |  |
|  | Not submitted (*Sarcocystis* sp. E389-13A-28S) | 142 | *Maxomys whiteheadi*  (Borneo) | n.a. | *versus* *Sarcocystis* sp. E369-13-28S, identities: 142/142 (100%), gaps: 0/142 (0%); | Sarcocyst identified (Fig 2a, b) |
|  |  |  |  |  | *versus Sarcocystis* sp. Rn-cl1.3-28S, identities: 142/142 (100%), gaps: 0/142 (0%) |  |
|  |  |  |  |  | *versus* Rn-cl2.3-28S, same as above |  |
|  |  |  |  |  | *versus* KU341120: identities: 141/142 (99.30%), gaps: 0/142 (0%); |  |
|  | Not submitted (*Sarcocystis* sp. E389-13B-28S) | 137 | *Maxomys whiteheadi*  (Borneo) | n.a. | *versus Sarcocystis* sp. E389-13A-28S: identities: 137/137 (100%), gaps: 0/137 (0%) | Sarcocyst identified (Fig 2a, b) |
|  | OR979644 (*S.* cf. *scandentiborneensis* E388-13A-28S) | 413 | *Niviventer cremoriventer* (Borneo) | .n.a. | *versus* *Sarcocystis* sp. E369-13-28S, identities: 409/413 (99.03%), gaps: 0/413 (0%); | Sarcocyst morphology (H&E histology, TEM) similar to, but not unequivocally *S. scandentiborneensis*; genetically close to *S. scandentiborneensis* but sequence too short for conclusion on species. |
|  |  |  |  |  | *versus Sarcocystis* sp. Rn-cl2.3-28S, identities: 409/413 (99.03%), gaps: 0/413 (0%), |  |
|  |  |  |  |  | *versus* KU341120: identities: 407/413 (98.55%), gaps: 0/413 (0%) |  |
|  |  |  |  |  | *versus* *S. scandentiborneensis* E357-13-28S: identities: 412/413 (99.76%), gaps: 0/413 (0%) |  |
|  |  |  |  |  | *versus* *S. zuoi* Rn-cl2-3-28S, identities: 288/295 (97.63%). Gaps: 0/295 (0%) |  |
|  | OR979645 (*S.* cf. *scandentiborneensis* E367-13-28S | 614 | *Sundasciurus lowii* (Borneo) | n.a. | *versus Sarcocystis* sp. Rn-cl2.3-28S, identities: 607/614 (98.86%), gaps: 0/614 (0%) | Sarcocyst identified: LM and TEM of sarcocyst wall similar to *S. scandentiborneensis*; best match with this species in pairwise BLAST comparison |
|  |  |  |  |  | *versus* KU341120: Identities: 605/614 (98.53%), gaps: 0/614 (0%) |  |
|  |  |  |  |  | *versus* *S. scandentiborneensis* E357-13-28S, identities: 546/547 (99.82%), gaps 0/547 (0%) |  |
|  |  |  |  |  | *versus* *S*. *zuoi* Rn-cl2-3-28S, identities: 442/445 (97.14%), gaps: 3/455 (0%) |  |
|  | OR979646 (*S.* cf. *scandentiborneensis* E346-13-28S) | 598 | *Sundasciurus lowii* | n.a. | *versus Sarcocystis* sp. Rn-cl2.3-28S, identities: 591/598 (98.83%), gaps: 0/598 (0%) | Sarcocyst identified: LM and TEM of sarcocyst wall similar to *S. scandentiborneensis*; best match with this species in pairwise BLAST comparison |
|  |  |  |  |  | *versus* KU341120, identities: 589/598 (98.49%), gaps: 0/598 (0%) |  |
|  |  |  |  |  | *versus* *S. scandentiborneensis* E357-13-28S, identities: 546/547 (99.82%), gaps: 0/547 (0%) |  |
|  |  |  |  |  | *versus* *S*. *zuoi* Rn-cl2-3-28S, identities: 426/439 (97.04%), gaps: 3/439 (0%) |  |
|  | OR979647 (*S. scandentiborneensis* E357-13-28S) | 547 | *Tupaia minor* (Borneo) | n.a. | *versus Sarcocystis* sp*.* Rn-cl2.3-28S, identities: 541/547 (98.90%), gaps: 0/547 (0%) | Corresponding sarcocyst known: *S. scandentiborneensis* [20] |
|  |  |  |  |  | *versus* KU341120: identities: 539/547 (98.54%), gaps 0/547 (0%) |  |
|  |  |  |  |  | *versus* *S*. *zuoi* Rn-cl2-3-28S, identities: 380/388 (97.94%), gaps: 0/388 (0%) |  |
|  | OR979648 (*S. zuoi* Rn-cl2.3-28S) | 3150 | *Rattus norvegicus* (China) | n.a. | *versus* *Sarcocystis* sp. Rn-cl1.3-28S, identities: 3081/3162 (97.44%), gaps: 35/3162 (1%) | Sarcocyst described by Hu et al. [13] |
|  |  |  |  |  | *versus* *Sarcocystis* sp. Rn-cl2.3-28S, identities: 3082/3166 (97.35%), gaps: 39/3166 (1%) |  |
|  |  |  |  |  | *versus* *Sarcocystis* sp. Rn-cl3.1-28S, identities: 3078/3159 (97.44%), gaps: 30/3159 (0%) |  |
|  |  |  |  |  | *versus* *Sarcocystis* sp. Rn-cl3.3-28S, identities: 3082/3162 (97.47%), gaps: 36/3162 (1%) |  |
|  |  |  |  |  | *versus Sarcocystis* sp. E369-13-28S (Borneo), identities: 440/452 (97.35%), gaps: 1/452 (0%) |  |
| ITS1*-5.8S-*ITS2 | OR977565 (*Sarcocystis* sp. Cl312-ITS/5.8S) | 1282 | *Rattus norvegicus* (China) | 97.0 – 99.0 (average 98.0) | *versus* KU341120: identities: 1188/1276 (93.10%), gaps: 36/1276 (2%) | Sarcocyst identified (Fig 2d, e) |
|  |  |  |  |  | *versus* *S*. *attenuati* MZ826990, identities: 655/734 (89.24%), gaps: 34/734 (4%) |  |
|  |  |  |  |  | *versus S*. *zuoi* (see below) |  |
|  | OR977566 (*Sarcocystis* sp. Cl41-ITS/5.8S) | 1256 | *Rattus norvegicus* (China) | 97.0 – 99.0 (average 98.0) | *versus* KU341120, identities: 1165/1268 (91.88%), gaps: 46/1268 (3%) | Sarcocyst identified (Fig 2d, e) |
|  |  |  |  |  | *versus* *S. attenuati* MZ826990, identities: 636/712 (89.33%), gaps: 34/712 (4%) |  |
|  | OR977567 (*Sarcocystis* sp. Cl43-ITS/5.8S) | 1263 | *Rattus norvegicus* (China) | 97.0 – 99.0 (average 98.0) | *versus* KU341120: identities: 1171/1269 (92.28%), gaps: 41/1269 (3%) | Sarcocyst identified (Fig 2d, e) |
|  |  |  |  |  | *versus* *S*. *attenuati* MZ826990, identities: 652/734 (88.83%), gaps: 36/734 (4%) |  |
|  | OR977568 (*Sarcocystis* sp. Cl45-ITS/5.8S) | 1263 | *Rattus norvegicus* (China) | 97.0 – 99.0 (average 98.0) | *versus* KU341120: identities: 1168/1273 (91.75%), gaps: 46/1273 (3%) | Sarcocyst identified (Fig 2d, e) |
|  |  |  |  |  | *versus* *S*. *attenuati* MZ826990, identities: 655/736 (88.99%), gaps: 37/736 (5%) |  |
| *Cox1* | PP033596  (*Sarcocystis* sp. 11526FR1-Cox1) | 1315 | *Rattus norvegicus* (China) | 100 | *versus* *S. attenuati* (MZ889673): identities:1328/1333 (99.62 %), gaps: 0/1333 (0%) | Sarcocyst identified (Fig 2d, e); three other, identical haplotypes not submitted. |
|  |  |  |  |  | *versus S. kani* (ON989199), identities: 924/927 (99.68%), gaps: 0/927 (0%) |  |
|  |  |  |  |  | *versus* *S. scandentiborneensis* (MN732562), identities: 973/976 (99.69 %), gaps: 0/976 (0%) |  |
|  |  |  |  |  | *versus* *S. zuoi* SF1-Rm1011-Cox1, identities: 970/978 (99.18 %), gaps: 0/978 (0%) |  |
|  |  |  |  |  | *versus Sarcocystis* sp.2, Sumatra, (ON989200), identities: 966/986 (97.97%), gaps: 0/986 (0%) |  |
|  |  |  |  |  | *versus S*. *singaporensis* (ON989197), identities: 931/994 (93.66%), gaps: 0/944 (0%) |  |
|  |  |  |  |  | *versus S*. *zamani* (ON989198), identities: 912/955 (95.50%), gaps: 0/955 (0%) |  |
|  |  |  |  |  | *versus* *S. pantherophisi* (KU891603), identities: 964/1015 (94.98%), gaps: 0/1015 (0%) |  |
|  | PP033597 (*Sarcocystis zuoi* SF1-Rm1011-Cox1) | 978 | *Rattus norvegicus* (China) | n.a. | *versus* *S. attenuati* (MZ889669): identities: 973/978 (99.49%), gaps: 0/978 (0%) | Sarcocyst of *S. zuoi* [13] |
|  |  |  |  |  | *versus S. kani* (ON989199), identities: 922/927 (99.49%), gaps: 0/927(0%) |  |
|  |  |  |  |  | *versus* *S. scandentiborneensis* (MN732561), identities: 936/939 (99.68%), gaps: 0/939 (0%) |  |
|  |  |  |  |  | *versus* *Sarcocystis* sp. 11526FR1-Cox1 (see above) |  |
|  |  |  |  |  | *versus* *S. singaporensis* (ON989197), identities: 876/934 (93.79%), gaps: 0/934 (0%) |  |
|  |  |  |  |  | *versus S*. *zamani* (ON989198), identities: 892/934 (95.50%), gaps: 0/934 (0%) |  |
|  |  |  |  |  | *versus* *S. pantherophisi* (KU891603), identities: 930/978 (95.09%), gaps: 0/978 (0%) |  |

All similarity comparisons were performed using the web-based Basic Local Alignment Search Tool (BLAST algorithm) of the National Center for Biotechnology Information of the National Institutes of Health, USA. The ribosomal RNA gene sequences under accession number KU341120 (‘*Sarcocystis* *zuoi*’: *18S*, *28S* and ITS1*-5.8S-*ITS2) often appeared as best match in BLAST searches for *Sarcocystis* sp., which is why they are frequently referenced here; note that we excised single gene partitions for the purpose of comparison; the same applies to the related sequences KU341118/19/21. Information on the position of the eukaryotic expansion segments (ES) of the *28S rRNA* gene is based on the predicted secondary structure of the orthologous sequence of *Toxoplasma gondii* as published by Gagnon et al. [26]. The sequences of the ITS1*-5.8S-*ITS2 gene complex were trimmed to their boundaries.

n.a. = not applicable

^a^According to the predicted secondary structure of the 28S rRNA of *Toxoplasma gondii* [26]
